# HIF-1α participates in the regulation of S100A16-HRD1-GSK3β/CK1α pathway in renal hypoxia injury

**DOI:** 10.1038/s41419-024-06696-5

**Published:** 2024-05-06

**Authors:** Shuying Han, Runbing Jin, Lei Huo, Yunfei Teng, Lihua Zhao, Kaini Zhang, Rongfeng Li, Dongming Su, Xiubin Liang

**Affiliations:** 1 Department of Pathophysiology, Nanjing Medical University, Nanjing, 211166 China; 2 Department of Pathology, Union Hospital, Tongji Medical College, Huazhong University of Science and Technology, Wuhan, Hubei 430022 China; 3 Department of Pathology, Nanjing Medical University, Nanjing, 211166 China; 4 Jiangsu Key Laboratory of Xenotransplantation, Nanjing Medical University, Nanjing, 211166 China

**Keywords:** Kidney diseases, Cell signalling

## Abstract

S100 calcium-binding protein 16 (S100A16) is implicated in both chronic kidney disease (CKD) and acute kidney injury (AKI). Previous research has shown that S100A16 contributes to AKI by facilitating the ubiquitylation and degradation of glycogen synthase kinase 3β (GSK3β) and casein kinase 1α (CK1α) through the activation of HMG-CoA reductase degradation protein 1 (HRD1). However, the mechanisms governing S100A16-induced HRD1 activation and the upregulation of S100A16 expression in renal injury are not fully understood. In this study, we observed elevated expression of Hypoxia-inducible Factor 1-alpha (HIF-1α) in the kidneys of mice subjected to ischemia-reperfusion injury (IRI). S100A16 deletion attenuated the increased HIF-1α expression induced by IRI. Using a S100A16 knockout rat renal tubular epithelial cell line (NRK-52E cells), we found that S100A16 knockout effectively mitigated apoptosis during hypoxic reoxygenation (H/R) and cell injury induced by TGF-β1. Our results revealed that H/R injuries increased both protein and mRNA levels of HIF-1α and HRD1 in renal tubular cells. S100A16 knockout reversed the expressions of HIF-1α and HRD1 under H/R conditions. Conversely, S100A16 overexpression in NRK-52E cells elevated HIF-1α and HRD1 levels. HIF-1α overexpression increased HRD1 and β-catenin while decreasing GSK-3β. HIF-1α inhibition restored HRD1 and β-catenin upregulation and GSK-3β downregulation by cellular H/R injury. Notably, Chromatin immunoprecipitation (ChIP) and luciferase reporter assays demonstrated HIF-1α binding signals on the HRD1 promoter, and luciferase reporter gene assays confirmed HIF-1α‘s transcriptional regulation of HRD1. Additionally, we identified Transcription Factor AP-2 Beta (TFAP2B) as the upregulator of S100A16. ChIP and luciferase reporter assays confirmed TFAP2B as a transcription factor for S100A16. In summary, this study identifies TFAP2B as the transcription factor for S100A16 and demonstrates HIF-1α regulation of HRD1 transcription within the S100A16-HRD1-GSK3β/CK1α pathway during renal hypoxia injury. These findings provide crucial insights into the molecular mechanisms of kidney injury, offering potential avenues for therapeutic intervention.

## Introduction

S100A16 is a member of the S100 protein family, a group of small, calcium-binding proteins that play crucial roles in various cellular processes, including cell proliferation, differentiation, and inflammation [1, 2]. The S100A16 gene is located on human chromosome 2q37.1 and encodes a protein of approximately 106 amino acids. Its expression has been detected in multiple tissues where it appears to participate in the regulation of cellular functions [3].

Among the S100 family members, S100A16 has garnered increasing attention in recent years due to its potential involvement in disease pathogenesis. Notably, S100A16 has been implicated in various disease processes, including cancer, inflammation, metabolic disorders, and kidney diseases [4–7]. For instance, in the context of cancer, altered S100A16 expression has been associated with tumor development, progression, and metastasis in certain cancer types. Additionally, investigations into its role in inflammatory diseases have suggested that S100A16 may play a part in promoting pro-inflammatory responses and tissue damage [8, 9]. Furthermore, there are indications that S100A16 might be involved in metabolic regulation and could potentially impact the pathogenesis of metabolic disorders [10, 11].

In recent studies using animal models, researchers have explored the potential implications of S100A16 in kidney disease and renal function. One study demonstrated a significant increase in S100A16 expression in the kidneys of diabetic mice, suggesting a possible involvement in promoting kidney inflammation and fibrosis, key factors contributing to diabetic nephropathy [10]. In our previous studies, we reported that the protein expression of S100A16 is significantly increased in the kidney of unilateral ureteral occlusion (UUO) mice, and the characteristic pathological changes of renal tubulointerstitial fibrosis appeared in the kidney of S100A16 transgenic mice, indicating a positive relationship between S100A16 and tubulointerstitial fibrosis. Moreover, S100A16 is also highly expressed in kidney biopsy specimens from patients with various clinical nephropathy [12]. Using the S100A16 knockout mice under IRI conditions, we have also identified the S100A16-HRD1-GSK3β/CK1α axis as a new Wnt/β-catenin signaling mechanism [7] in AKI. We reported that S100A16 activated HRD1, an E3 ubiquitin ligase, that degraded GSK3β and CK1α, the major members of the β-catenin degradation complex, then allowed β-catenin to release and translocate to the nucleus, leading to severe kidney damage. Our studies suggest that the S100A16 is upregulated and served as a significant regulator when kidney injury occurs; however, the mechanism of why S100A16 induced HRD1 upregulation, and why the expression of S100A16 is increased in renal injury, have yet to be fully elucidated.

HRD1 (HMG-CoA reductase degradation protein 1), also known as SYVN1 (Synoviolin-1), is an E3 ubiquitin ligase that plays a crucial role in the endoplasmic reticulum (ER)-associated degradation (ERAD) pathway [13, 14]. The transcriptional regulation of HRD1 is a complex process, and its expression can be modulated by various factors and signaling pathways. Several transcription factors have been identified as regulators of HRD1 gene expression. Hypoxia-inducible factor-1 alpha (HIF-1α) is a transcription factor that plays a critical role in cellular responses to hypoxia [15]. In normoxic conditions, HIF-1α is continuously produced but rapidly degraded by the proteasome. However, under hypoxic conditions, HIF-1α degradation is suppressed, allowing it to accumulate and translocate to the nucleus, where it binds to hypoxia-responsive elements (HREs) in the promoter regions of target genes, leading to their transcriptional activation [16]. These target genes include various molecules involved in adaptive responses to hypoxia, such as those regulating angiogenesis, erythropoiesis, glucose metabolism, and AKI. While the role of HIF-1α in AKI has been extensively studied, and it has been implicated in the regulation of numerous genes in response to hypoxia, its direct involvement in HRD1 expression in the context of AKI has not been reported. After prediction analysis, we found the promoter of HRD1 includes HRES. Therefore, we hypotheses that there is a direct linking HIF-1α to HRD1 regulation in AKI.

Transcription Factor AP-2 Beta (TFAP2B) is a member of the AP-2 family of transcription factors. This family includes five closely related members (AP-2α, AP-2β, AP-2γ, AP-2δ, and AP-2ε) that share a high degree of sequence homology and similar DNA-binding properties [17, 18]. TFAP2B is expressed in various tissues during development and has been implicated in various biological processes, including embryonic development, cell proliferation, differentiation, and tissue-specific gene expression [19, 20]. To explore the main mechanism of up-regulation of S100A16 expression, we used relevant websites to predict targeted transcription factors of S100A16 promoter and analyzed the expression of predicted transcription factors in the UUO model and biopsy samples from CKD patients by the GEO database. The results suggest that TFAP2B is a candidate for S100A16 transcription factors. In this study, we tested the transcript regulation of TFAP2B on S100A16 using HK-2 cells.

Our current study aims to determine if HIF-1α regulates HRD1 transcription in S100A16-HRD1-GSK3β/CK1α pathway during renal hypoxia injury, and if TFAP2B is S100A16 transcription factor.

## Results

### S100A16 knockout decreases HIF-1α expression in AKI mice

In our previous study, we reported that S100A16 promotes acute kidney injury (AKI) by activating E3 ubiquitin ligase, the HMG-CoA reductase degradation protein 1 (HRD1), induced ubiquitination and degradation of GSK3β and CK1α, two β-catenin complex members [7]. β-catenin is subsequently released in response to AKI. However, it is unknown why HRD1 is increased in AKI. Hypoxia-inducible Factor 1-alpha (HIF-1α) is a transcription factor that plays a crucial role in the regulation of cellular responses to hypoxia. In this study, the renal ischemia-reperfusion injury (IRI) model in S100A16 knockout mice (S100A16^+/−^) was used to determine if HIF-1α is associated with HRD1 upregulation in AKI.

**Fig. 1:**
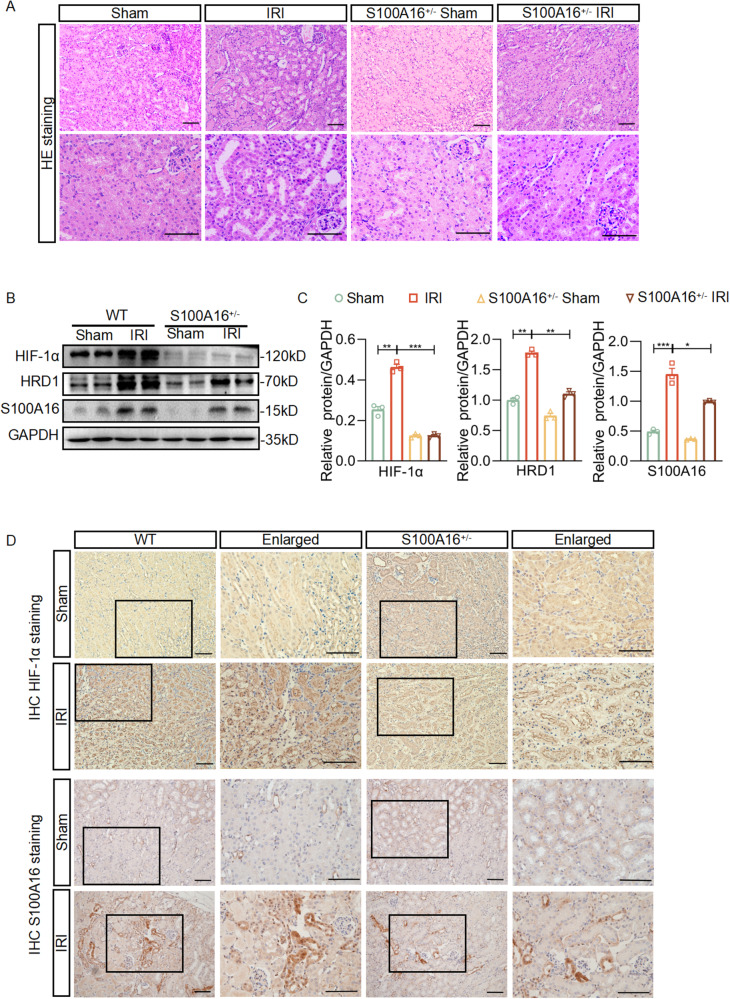
S100A16 knockout decreases HIF-1α expression in AKI mice. **A** HE staining in kidney sections from the WT mice and S100A16^+/−^ mice after IRI. **B** Western blots of HIF-1α, HRD1 and S100A16 in kidney tissues from WT mice and S100A16^+/−^ mice after IRI. **C** Quantitation of western blot data for HIF-1α, HRD1 and S100A16 in **B**. ***P < 0.0001, **P < 0.01, *P < 0.05. **D** Immunohistochemical staining of HIF-1α and S100A16 in the WT mice and S100A16^+/−^ mice after IRI. Scale bar: 50 μm.

The histological results in mouse kidneys by hematoxylin-eosin (HE) staining demonstrated that IRI-induced kidney injury, including tubular dilatation and glomerular atrophy. S100A16 knockout attenuated IRI in mice (Fig. 1A). As shown in Fig. S1A, S100A16^+/−^ mice presented a significantly decreased pathologic score on the tubular injury compared with WT mice after IRI induction. These data were consistent with our previous studies [7]. The expressions of S100A16 and HRD1 detected by western blots were significantly increased in the IRI group compared to the sham-operated group in WT mice (Fig. 1B,C), which are consistent with our previous reports [7]. Interestingly, we also observed that the expression of HIF-1α was mainly expressed in renal tubular epithelial cells, and significantly increased in the kidneys in the IRI mice compared to the sham-operated group (Fig. 1D). S100A16 knockout attenuated the increased expression of HIF-1α induced by the IRI (Fig. 1B,C). The results of immunohistochemical (IHC) staining in HIF-1α, S100A16 and HRD1 expressions are also consistent with the biochemistry data (Fig. 1D, Figs. S1B and S1C). The reciprocal relationship of HIF-1α and S100A16 in IRI mice led us to hypothesize that S100A16 upregulated HRD1 level through HIF-1α activation in renal injury conditions.

### S100A16 knockout attenuates the injuries induced by hypoxic reoxygenation and TGF-β1 stimulation in NRK-52E cells

**Fig. 2:**
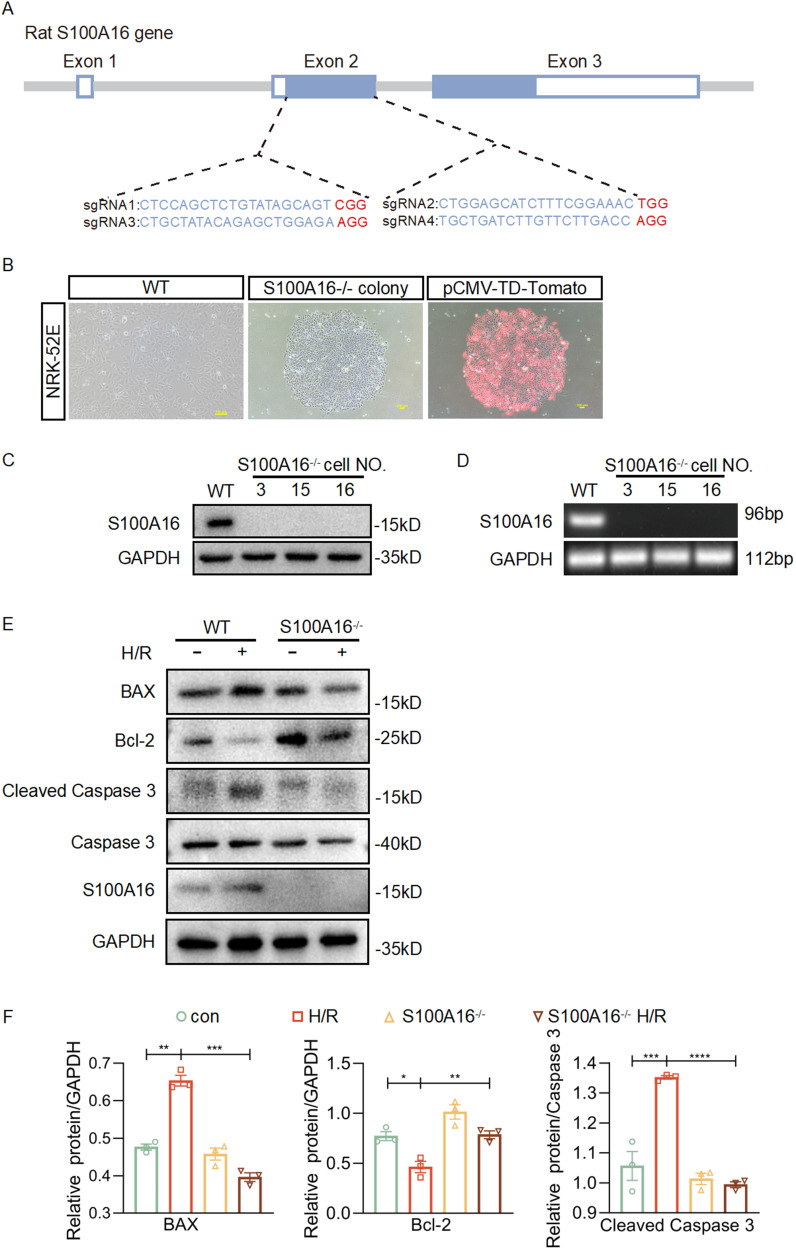
S100A16 knockout attenuates the injuries induced by hypoxic reoxygenation in NRK-52E cells. **A** Four sgRNAs were designed at the anterior and posterior end of the second exon of S100A16 for the generation of S100A16 knockout in NRK-52E cells. **B** Monoclonal cell clusters formed after 7–10 days of G418 drug selection. **C** Three S100A16^−/−^ monoclonal cell lines were selected to validate S100A16 protein by western blot. **D** RT-PCR was performed to check mRNA levels in three S100A16^−/−^ monoclonal cell lines. **E** Western blots of BAX, Bcl-2, Cleaved Caspase3, Caspase3, and S100A16 in S100A16^−/−^ cells. **F** Quantitation of western blot data for BAX, Bcl-2, and Cleaved Caspase3 in **E**. ****P < 0.0001, ***P < 0.001, **P < 0.01, *P < 0.05.

To further determine whether S100A16 modulates HIF-1α expression in renal injury, we used CRISPR/Cas9 gene editing technology to knockout S100A16 in rat renal tubular epithelial cells (NRK-52E cells). After analyzing rat derived S100A16 gene (Gene ID: 361991) in GENEBANK, four sgRNAs pairs in the second S100A16 exon were designed using the online design tool (https://www.zlab.bio/resources) (Fig. 2A). The four pairs of sgRNAs were ligated to the PX330 plasmid to form the ligation product S100A16-Cas9/sgRNA. After sequencing and identification, the four pairs of ligation products were transfected into NRK-52E cells for T7E1 cleavage assay and sequence analysis. The sgRNA cleavage efficiency is shown in Fig. S2A. sgRNA3>sgRNA1>sgRNA4>sgRNA2, consistent with the results of website analysis (https://tider.deskgen.com/) (Fig. S2B). The sgRNAs with high cleavage efficiency (sgRNA3 and sgRNA4) and pCMV-TD-Tomato were selected for transfection into NRK-52E cells in a 5:1 ratio, and the transfected cells were treated with G418 (Fig. 2B) with 19 positive monoclonal drugs screened clones from 33 monoclonal cell lines (Fig. S2C). Three S100A16^−/−^ cell clones were selected for validation at the protein (Fig. 2C) and mRNA levels of S100A16 (Fig. 2D). S100A16^−/−^ cells were also confirmed by cellular immunofluorescence (Fig. S2D). Compared to WT cells, S100A16^−/−^ cells have no difference in cell morphology (Fig. S3A) and growth viability (Fig. S3B). Western Blot analysis showed that S100A16 knockout effectively inhibited H/R-induced upregulation of pro-apoptotic genes, including BAX, Cleaved Caspase3 and Caspase3, and downregulation of Bcl-2 (Fig. 2E,F). TUNEL staining assay also confirmed that S100A16 knockout significantly attenuated H/R-induced cell apoptosis (Fig. S3C). Furthermore, S100A16 knockout suppressed the TGF-β1-induced upregulation of Fibronectin, α-SMA expression, an indicator of renal fibrosis in NRK-52E cells (Figs. S3D and E). These data show that knockout of S100A16 can effectively attenuate apoptosis caused by hypoxic reoxygenation and TGF-β1-induced cell injury.

### S100A16 modulates the expression of HIF-1α and HRD1 in NRK-52E cells

**Fig. 3:**
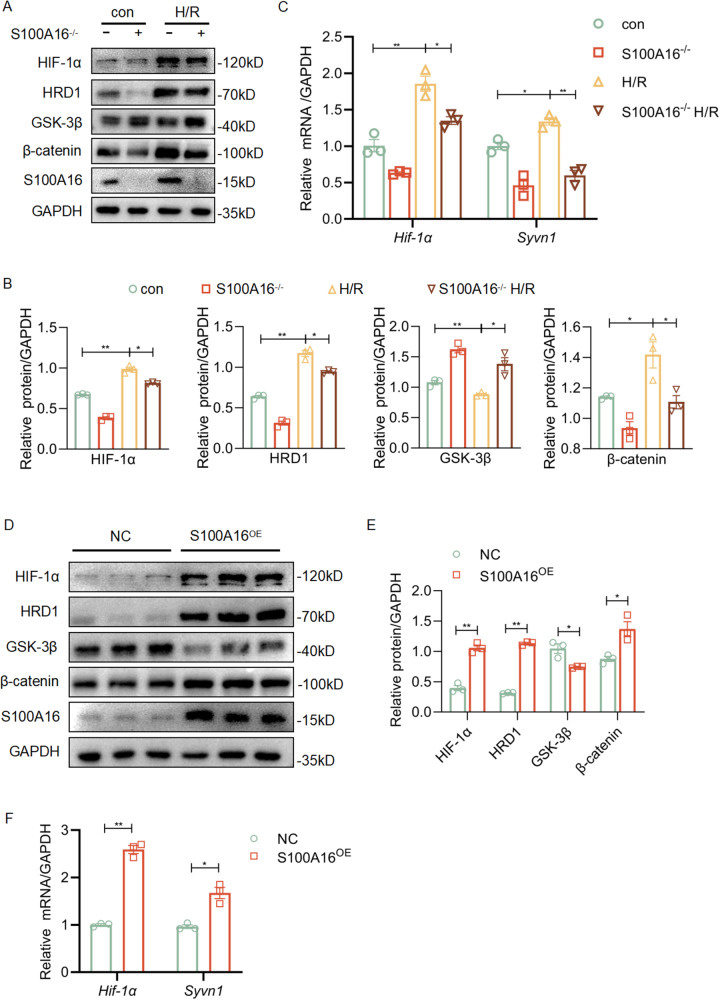
S100A16 modulates the expression of HIF-1α and HRD1 in NRK-52E cells. **A** Western blots of HIF-1α, HRD1, GSK-3β, β-catenin, and S100A16 in normal or H/R conditions using S100A16^−/−^ cells. **B** Quantitation of western blot data for HIF-1α, HRD1, GSK-3β, and β-catenin in **A**. **P < 0.01, *P < 0.05. **C** The transcriptional expressions of *Hif-1α* and *Syvn1* were tested by real-time PCR in normal or H/R conditions using the S100A16^−/−^ cell line. **P < 0.01, *P < 0.05. **D** Western blots of HIF-1α, HRD1, GSK-3β, β-catenin, and S100A16 in NRK-52E cells overexpressed with S100A16. **E** Quantitation of the expression of HIF-1α, HRD1, GSK-3β, and β-catenin in **D**. **P < 0.01; *P < 0.05. **F** The transcriptional expressions of *Hif-1α* and *Syvn1* were tested by real-time PCR in S100A16 overexpressing NRK-52E cells. **P < 0.01; *P < 0.05.

Next, using S100A16^−/−^ cells, we determined if S100A16 regulated HIF-1α and HRD1 with Wnt/β-catenin signaling pathway in a cellular hypoxia-reoxygenation (H/R) model. Our results revealed that H/R injuries significantly increased both protein and mRNA levels of HIF-1α, HRD1 and β-catenin, decreased GSK-3β in NRK-52E cells. However, the knockout of S100A16 significantly reversed the expressions of HIF-1α, HRD1, β-catenin and GSK-3β in S100A16^−/−^ cells under the H/R conditions (Fig. 3A, B). Similarly, the transcript levels of *Hif-1α* and *Syvn1* were all upregulated in NRK-52E cells under the H/R conditions (Fig. 3C).

In contrast, S100A16 overexpression in NRK-52E cells by the transient transfection with S100A16 plasmid increased the expressions of HIF-1α, HRD1, and β-catenin, decreased GSK-3β protein level (Fig. 3D,E). The transcript levels of *Hif-1α* and *Syvn1* were all upregulated that consistent with their protein expressions (Fig. 3F). The changes of HRD1, β-catenin and GSK-3β in NRK-52E cells transfected with S100A16 consistent with our previous reports [7]. Our concept is that HRD1 can ubiquitously degrade GSK-3β, thus allowing cytoplasmic β-catenin accumulation into the nucleus to initiate transcription of downstream genes. However, if using BAY, a HIF-1α inhibitor, to inhibit HIF-1α expression in NRK-52E cells, we found that HRD1 upregulation caused by S100A16 overexpression was markedly blunted (Fig. S4A,B). Therefore, we assumed that the upregulation of HRD1 was a result of HIF-1α, which was growing in H/R-damaged NRK-52E cells.

### HIF-1α transcriptionally upregulates HRD1 expression in HK-2 cells

**Fig. 4:**
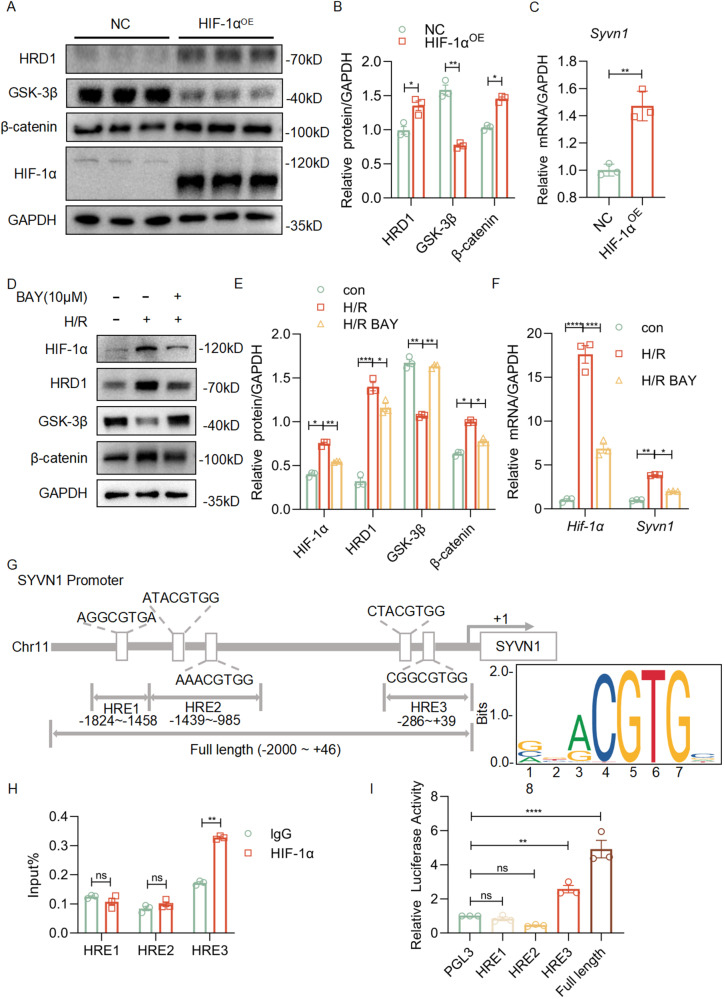
HIF-1α transcriptionally upregulates HRD1 expression in HK-2 cells. **A** Western blots of HRD1, GSK-3β, β-catenin and HIF-1α in HK-2 cells overexpressed by HIF-1α. **B** Quantitation of the expression of HRD1, GSK-3β and β-catenin in **A**. **P < 0.01; *P < 0.05. **C** The transcriptional expressions of *SYVN1* were tested by real-time PCR in HIF-1α overexpressing HK-2 cells. **P < 0.01; HIF-1α^OE^ vs. NC. **D** Western blots of HIF-1α, HRD1, GSK-3β, and β-catenin in HK-2 cells treated with or without BAY. **E** Quantitation of western blot data for HIF-1α, HRD1, GSK-3β, and β-catenin in **D**. ***P < 0.001, **P < 0.01, *P < 0.05. **F** The transcriptional expressions of *HIF1A* and *SYVN1* were tested by real-time PCR in HK-2 cells treated with or without BAY. ****P < 0.0001, ***P < 0.001, **P < 0.01, *P < 0.05. **G** Analysis of the potential binding sites of the *SYVN1* (HRD1) promoter with HIF-1α according to the jaspar website. **H** ChIP assay was performed to determine the binding of HIF-1α to the SYVN1 promoter. **P < 0.01, ns: no significant; vs. IgG. **I** Luciferase activity analysis in HK-2 cells transfected with different reporter luciferase plasmids. ****P < 0.0001, **P < 0.01, ns: no significant; vs. PGL3.

To clarify the relationship between HIF-1α and HRD1, we used human kidney-2 (HK-2) cells, which are human renal proximal tubular epithelial cells to perform the experiments of HIF-1α overexpression. We transfected the HIF-1α plasmid into HK-2 cells. The results revealed that overexpression of HIF-1α significantly upregulated HRD1 and β-catenin, and downregulated GSK-3β (Fig. 4A,B). At the mRNA level, overexpression of HIF-1α, the expression of *SYVN1* was significantly increased (Fig. 4C). In the cellular H/R model, inhibition of HIF-1α expression by BAY reversed the upregulation of HRD1 and β-catenin expression and the downregulation of GSK-3β by cellular H/R injury (Fig. 4D,E). The mRNA level of *SYVN1* was suppressed after inhibition of HIF-1α expression using BAY in the cellular H/R model (Fig. 4F). The results suggest that HIF-1α directly regulates the transcription of HRD1.

We hence hypothesized that HIF-1α is a transcription factor of SYVN1 (HRD1). To test this, we analyzed the potential binding sites of the HRD1 promoter with HIF-1α at the website https://jaspar.genereg.net/. We identified five potential binding sites in the HRD1 promoter (Supplementary Table 1.1). We designed three regions at the −1824 ~ −1458 bp (HRE1), −1439 ~ −985 bp (HRE2), −286 ~ +39 bp (HRE3) position of the HRD1 gene to determine the interaction between HRD1 promoter and HIF-1α (Fig. 4G) [16]. Chromatin immunoprecipitation (ChIP) assay revealed that there are HIF-1α binding signals on the HRD1 promoter region at the HRE3 area in the HK-2 cells (Fig. 4H).

To test if HIF-1α binds to this sequence to exert its transcriptional regulation of HRD1, we constructed the reporter plasmid consisting of the HRD1 promoter sequence HRE1, HRE2, HRE3, and full length (−2000 ~ +46 bp), followed by a luciferase coding sequence (HRD1-Luc). We transfected the HK-2 cells with HRD1-Luc or empty vector, followed by transfection of these cells with HIF-1α plasmid. The relative luciferase signals from each of the combinations in HK-2 cells were quantified. As expected, HIF-1α increased the luciferase signal levels in HK-2 cells that were transfected with HRD1(HER3)-Luc (Fig. 4I). The data indicated that the HIF-1α’s binding to the HRD1 promoter induced the level of transcription of HRD1.

### TFAP2B and S100A16 are upregulated in injured HK-2 cells and UUO mouse kidney

In our previous studies, we reported that the expression of S100A16 is significantly increased in kidney biopsy specimens from patients with various clinical nephropathy and kidney disease mouse models, including IRI model and unilateral ureteral occlusion (UUO) model [6, 12]. However, the reasons why S100A16 highly expressed in renal disease are still unclear.

**Fig. 5:**
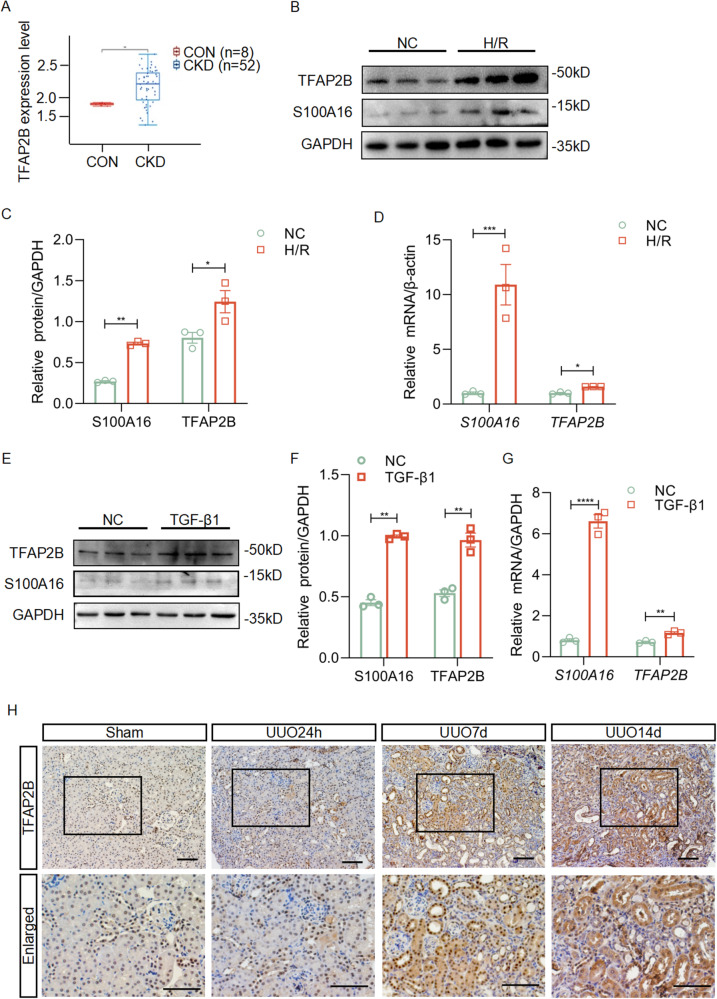
TFAP2B and S100A16 are upregulated in injured renal tubular cells and UUO mouse kidneys. **A** R2 genome analysis and visualization platform to determine TFAP2B expression levels in patients with CKD compared to normal controls using GEO dataset GSE66494. CON: normal kidney tissue; CKD: Kidney tissue of chronic kidney disease. **P < 0.01. **B** Western blots of TFAP2B and S100A16 in HK-2 cells with normal or H/R conditions. **C** Quantitation of western blot data for TFAP2B and S100A16 in **B**. **P < 0.01, *P < 0.05. **D** The transcriptional expressions of *S100A16* and *TFAP2B* were tested by real-time PCR in HK-2 cells with normal or H/R conditions. ***P < 0.001, *P < 0.05. **E** Western blots of TFAP2B and S100A16 in HK-2 cells treated with TGF-β1. **F** Quantitation of western blot data for TFAP2B and S100A16 in **E**. **P < 0.01. **G** The transcriptional expressions of *S100A16* and *TFAP2B* were tested by real-time PCR in HK-2 cells treated with TGF-β1. ****P < 0.0001, **P < 0.01. **H** Immunohistochemical staining of TFAP2B in mouse kidney after UUO. Scale bar: 50 μm.

To explore the mechanism of an increased S100A16 expression in kidney injury, we analyzed the differential expression of CKD samples (GSE66494) from the GEO database (Supplementary Table 2). The results revealed that the expression of TFAP2B, a transcript factor, was significantly upregulated in kidney biopsy tissues from patients with chronic kidney disease compared to healthy individuals (Fig. 5A). We examined the protein and transcript level of TFAP2B in HK-2 cells under the hypoxia-reoxygenation (H/R) injury. As shown in Fig. 5B,C, H/R injury significantly induced an increasing protein level of TFAP2B and S100A16 in HK-2 cells. Similarly, the transcript levels of *TFAP2B* and *S100A16* were all upregulated in injured HK-2 cells compared to normal control (Fig. 5D). However, TFAP2B knocking down in HK-2 cells by transient transfection of its siRNA reversed the upregulation of S100A16 induced by H/R injury (Fig. S5A,B). Using TGF-β1 treated cells, we also verified that TGF-β1 stimulation dramatically elevated the expression of TFAP2B and S100A16 at both protein levels (Fig. 5E,F) and mRNA levels (Fig. 5G). Consistently, TFAP2B protein expressions were higher in mice kidney tissues after UUO surgery detected by IHC staining (Fig. 5H and Fig. S5C), comparing with those in sham control subjects. These findings implied that TFAP2B regulates the transcription of S100A16 under renal injury.

### TFAP2B transcriptionally regulates S100A16 expression in HK-2 cells

**Fig. 6:**
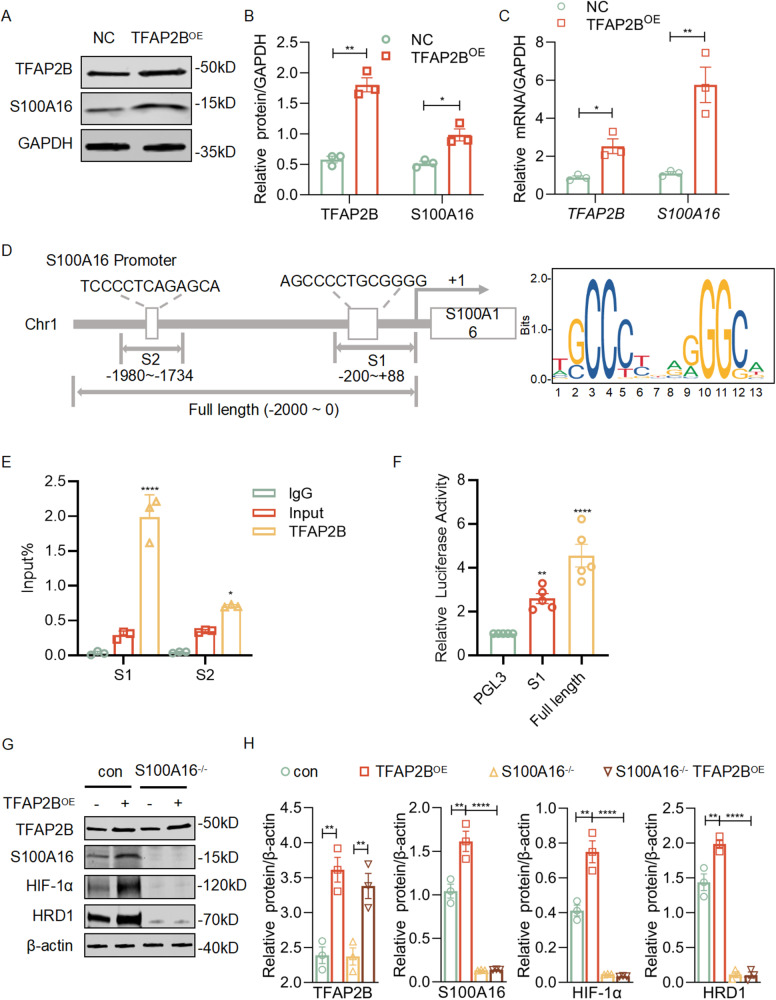
TFAP2B transcriptionally regulates S100A16 expression in HK-2 cells. **A** Western blots of TFAP2B and S100A16 in HK-2 cells overexpressed by TFAP2B. **B** Quantitation of western blot data for TFAP2B and S100A16 in **A**. **P < 0.01, *P < 0.05. **C** The transcriptional expressions of *TFAP2B* and *S100A16* were tested by real-time PCR in HK-2 cells overexpressed by TFAP2B. **P < 0.01, *P < 0.05. **D** Analysis of the potential binding sites of the S100A16 promoter with TFAP2B according to the jaspar website. **E** ChIP assay was performed to determine the binding of TFAP2B to the S100A16 promoter. ****P < 0.0001, *P < 0.05; vs IgG. **F** Luciferase activity analysis in HK-2 cells transfected with different reporter luciferase plasmids. ****P < 0.0001, **P < 0.01; vs. PGL3. **G** Western blots of TFAP2B, S100A16, HIF-1α and HRD1 in S100A16^−/−^ cells with or without TFAP2B overexpression. **H** Quantitation of western blot data for TFAP2B, S100A16, HIF-1α, and HRD1 in **G**. ****P < 0.0001, **P < 0.01.

We then asked the question of whether TFAP2B directly regulates the expression of S100A16. In HK-2 cells, overexpression of TFAP2B by its plasmid transfection resulted in significantly increased levels of S100A16 proteins (Fig. 6A,B) and transcripts (Fig. 6C). These results suggest TFAP2B directly regulates the transcription of S100A16 under the renal injury condition.

To test whether TFAP2B is a transcription factor of S100A16, we worked to determine if TFAP2B binds to the promoter of S100A16. First, we predicted the binding sites via the JASPAR website (Supplementary Table 1.2). The position of S100A16 promoter at −200 ~ +88 bp (S1) and −1980 ~ −1734 bp (S2) were two potential binding regions with TFAP2B (Fig. 6D). ChIP assay revealed that TFAP2B is bound to S1 and S2 region of S100A16 promoter, and there is a stronger binding of TFAP2B with S1 than that with S2 (Fig. 6E). To test if TFAP2B binding these regions to transcriptional regulate S100A16, the S100A16 promoter sequence at S1 (−200 ~ +88 bp) or Full length (−2000 ~ 0 bp) were ligated to the luciferase reporter gene constructs (S1-Luc, and Full length-Luc), which were further transfected into HK-2 cells with overexpressed TFAP2B plasmid. The relative luciferase signals from each of the combinations in HK-2 cells were quantified. The data showed that TFAP2B increased the luciferase signal levels in HK-2 cells that were transfected with S100A16-Luc (Fig. 6F). Finally, we found that TFAP2B overexpression in S100A16^−/−^ cells didn’t upregulate the expression of HIF-1α and HRD1 compared with that in control NRK-52E cells (Fig. 6G,H). These results suggest a TFAP2B-S100A16-HIF1α-HRD1 regulatory axis in renal tubular cells under the injured condition, and that TFAP2B directly regulates the transcription of S100A16.

## Discussion

S100A16 is predominantly localized in the cytoplasm of cells, where S100A16 can interact with a variety of target proteins, including signaling molecules and cytoskeletal components. These interactions can modulate cellular processes and affect various signaling pathways [21–23]. Some studies have suggested that S100A16 was involved in promoting cell proliferation, metastasis, and tumor growth in certain cancers, including prostate cancer and esophageal squamous cell carcinoma [24]. S100A16 is also viewed as a novel adipogenic factor involved in glycolipid metabolism [25]. S100A16 is also involved in the regulation of kidney disease or renal function. One study mentioned the increased expression of S100A16 in the kidneys of diabetic mice, suggesting a potential link to diabetic nephropathy [10]. In our previous studies, we reported that the expression of S100A16 is significantly increased in kidney biopsy specimens from patients with various clinical nephropathy and kidney disease mouse models, including IRI model and unilateral ureteral occlusion (UUO) model [12]. However, it is not known how S100A16 is activated in kidney injury. Here we provided evidence that TFAP2B is a transcription factor of S100A16.

TFAP2B (Transcription Factor AP-2 Beta) is a gene that encodes a member of the AP-2 family of transcription factors. It plays a significant role in various biological processes, including embryonic development, cell proliferation, differentiation, and apoptosis [26]. Regarding the kidney, TFAP2B is implicated in renal development and function. Specifically, TFAP2B has been found to be expressed in the developing kidney, where it participates in the formation of renal tubules and collecting ducts [18, 19]. It regulates the expression of genes involved in kidney development and function, such as those related to the epithelial-mesenchymal transition, nephron differentiation, and maintenance of the renal tubular architecture [27, 28]. Mutations in the TFAP2B gene have been associated with a genetic disorder called “Char syndrome” or “Lid2 syndrome.” This syndrome is characterized by facial dysmorphisms, heart defects, and patent ductus arteriosus [26]. Although the primary impact of TFAP2B mutations is on heart development, the gene’s expression and function in the kidney during embryonic development suggest a broader role in organogenesis [18]. Through analyzing the GEO database, we found that TFAP2B was significantly upregulated in kidney biopsy tissues from patients with chronic kidney disease. In this study, we identified that TFAP2B transcriptionally regulates S100A16 expression in renal tubular cells.

**Fig. 7:**
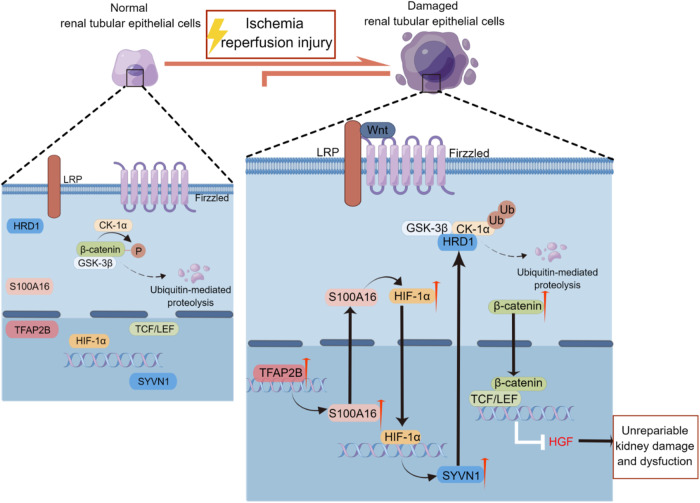
HIF-1α participates in the regulation of the S100A16-HRD1-GSK3β/CK1α pathway in renal hypoxiac injury. Under hypoxic conditions, such as ischemia-reperfusion injury in the kidney, TFAP2B transcriptionally upregulates S100A16 expression in renal tubular epithelial cells; S100A16 then increases HIF-1α expression. Upregulated HIF-1α can accumulate in the nucleus to influence the expression of E3 ubiquitin ligase HRD1. HRD1 can bind GSK3β/CK-1α and degrade them through the ubiquitin-proteasome pathway, releasing β-catenin and further activating Wnt/β-catenin signaling pathway, boosting transcriptional regulation of downstream target genes, and eventually leading to irreversible kidney impairment.

We previously reported that S100A16 is a significant regulator of the Wnt/β-catenin signaling activation during AKI. GSK3β and CK1α, as members of the β-catenin degradation complex, interact with HRD1, an E3 ubiquitin ligase, to target proteins for ubiquitination and degradation in conditions of renal injury [7]. β-catenin is subsequently released and translocated into the nucleus in response to Wnt/β-catenin signaling activation, and the expression of HGF is repressed, leading to severe kidney damage [29]. S100A16 knockout also relieves the degree of renal injury in vivo and reduces Wnt/β-catenin signaling activated by IRI. Therefore, we identified that the S100A16-HRD1-GSK3β/CK1α axis contributes to Wnt/β-catenin signaling activation in the renal injury [7]. However, the mechanism that S100A16 upregulated HRD1 when AKI occurs remains unclear. Our data in this study suggested that S100A16 modulates the expression of HIF-1α, and HIF-1α is a transcription factor of HRD1 (Fig. 7).

According to AKI, HIF-1α‘s role is complex. During hypoxic conditions, such as ischemia-reperfusion injury that often occurs in AKI, HIF-1α is stabilized and activates a wide range of genes involved in adaptive responses. HIF-1α promotes the expression of vascular endothelial growth factor (VEGF), which stimulates the formation of new blood vessels (angiogenesis) to improve oxygen delivery to the injured kidney tissue [30]. HIF-1α activates genes that enhance cell survival and inhibit apoptosis, helping to protect kidney cells from damage during hypoxic stress [31]. HIF-1α adjusts cellular metabolism by promoting glycolysis and suppressing oxidative phosphorylation, helping cells adapt to low oxygen levels [32]. However, there are controversies regarding the pathological roles of HIFs in kidney injury and repair [33]. It was reported that HIF-1α is implicated in regulating inflammation and fibrosis, which are important factors contributing to the progression of AKI [34]. In a study utilizing the hypoxia/reoxygenation model, researchers observed that persistent activation of HIF-1α significantly upregulated the expression of α-smooth muscle actin and downregulated the expression of E-cadherin [35]. This observation was consistently substantiated by the genetic ablation of proximal tubule epithelial HIF-1α, which impeded kidney fibrosis development in the UUO model [36]. Correspondingly, the genetic upregulation of HIF-1a in tubular epithelial cells through the deletion of von Hippel-Lindau tumor suppressor (VHL) exacerbated interstitial fibrosis in a 5/6 renal ablation model [37]. Furthermore, in rat models of angiotensin II-induced renal injury and chronic ischemic renal injury, the augmentation of fibrotic proteins (α-smooth muscle actin and collagen) was counteracted by HIF-1α [38, 39]. From a mechanistic perspective, HIF signaling may participate in the transcriptional control of fibrogenic genes, as well as engage in crosstalk with other pro-fibrotic signaling pathways, such as TGF-β, NF-κB, Notch, and the PI3K/Akt pathways [40]. Our data on the activation of the HIF-1α-HRD1-GSK3β/CK1α axis by S100A16 in renal injury provide an important clue for the pathological roles of HIFs in kidney injury.

In sum, S100A16 is an interesting protein that may have significant implications in renal disease. Further research is required to better understand its precise roles and potential therapeutic applications.

## Methods and Materials

### Animal model

Male C57BL/6 J mice (WT) and S100A16 knockout mice (S100A16^+/−^) (10–12 weeks old, weight 20–24 g) were purchased from the Model Animal Center of Nanjing University (contract number: (2009) T67). All animal experiments and maintenance protocols followed the principles of the Institutional Animal Protection and Use Committee of Nanjing Medical University. AKI model experiments were divided into four groups (10 mice per group): wild-type (C57BL/6) mice sham-operated group (WT Sham), wild-type (WT) mice renal ischemia-reperfusion (IRI sham) group, S100A16^+/−^ mice sham-operated group (S100A16^+/−^ Sham), and S100A16^+/−^ mouse renal ischemia-reperfusion (S100A16^+/−^ IRI) group. The experimental protocol for IRI surgery was as described previously [7]. Unilateral ureteral ligation (UUO) surgery was performed by ligating the left ureter on the kidney tissue of 12-week-old male C57BL/6 J mice. The UUO group and the contralateral kidney were removed 24 hours, 7 day, and 14 days after surgery, and a portion of the kidney was fixed using 10% formalin for histology and IHC staining. The other part of the kidney tissue was stored at −80 °C.

### Materials

Chemicals were purchased from Sigma-Aldrich (St. Louis, MO, USA) unless indicated otherwise. The assay kits and antibodies are presented in Supplementary Table 3.

### Cell culture, processing, and transfection

NRK-52E (Normal Rat Kidney-52E) were cultured in DMEM with 10% fetal bovine serum (FBS), and HK-2 (Human kidney-2) cells were cultured in DMEM/F12 containing 10% FBS at 37 °C, 5% CO_2_, 95% air. Anairopack was used to establish hypoxia/reoxygenation models for HK-2 and NRK-52E cells under hypoxia and reoxygenation for 24 hours. Under hypoxic/reoxygenated conditions, HK-2 cells were treated with 10 μM HIF-1α inhibitor BAY. After the cells were grown to 50–60%, growth was stopped for 8–12 h in a serum-free culture medium. Then treated with 20 ng/ml of TGF-β1 for 48 h to mimic renal fibrotic injury. The cells were transfected with the plasmids of S100A16, HIF-1α, TFAP2B, pcDNA3.1, and TFAP2B siRNA using transfection reagent followed by instructions. The sequences of the TFAP2B siRNA: Sense: 5-AGU UCA ACU UCG AAG UAC Att-3; antisense: 5-UGU ACU UCG AAG UUG AAC Uga-3 [41].

### Western blotting assay

Mouse kidney tissue or total cellular protein lysis products were separated by 8% or 12% sodium dodecyl sulfate-polyacrylamide gel electrophoresis (SDS-PAGE) and transferred to nitrocellulose membranes (PVDF). TBST buffer with 5% skim milk and 0.1% Tween-20 was closed at room temperature for 2 h. Primary antibodies were incubated overnight at 4 °C, followed by incubation of horseradish peroxidase-labeled secondary antibodies. Protein bands were detected with enhanced chemiluminescence solution (WestnBrightTM ECL) and images were obtained using a Tanon exposure meter and Image Quant ECL system (PerkinElmer Life Sciences, Wellesley, MA). Western blotting data were obtained using Image J software.

### Histological and immunohistochemical staining

Mouse kidney tissues were treated overnight in 4% paraformaldehyde, graded and dehydrated, embedded in wax blocks, and then cut into approximately 5 µm thick sections for HE staining and immunohistochemical (IHC) staining. Renal tissue sections were routinely stained with HE to observe histopathological changes in the kidney. Tubular injury score after IRI was determined by HE-stained sections according to the criteria: tubular dilation, brush border loss, tubular necrosis, and neutrophil infiltration in randomly chosen 3 high-power fields. Each field was scored following standard; 0, normal; 1, damage involving <25% of tubules; 2, damage involving 25–50% of tubules; 3, damage involving 50–75% of tubules; and 4, damage involving 75–100% of tubules [42].

For IHC staining, sections were placed in an oven at 65 °C for 30 min, 0.01 M citrate buffer (pH 6.0) and autoclave (95 °C, 2 min) to repair antigen, washed with phosphate-buffered saline (PBS) for 5 min × 3 times, and then incubated with primary antibody overnight at 4 °C. After washing with PBS for 5 min × 3 times, incubate with IHC secondary antibody at 37 °C for 30 min. Sections were stained with 3,3’-diaminobenzidine (DAB), counterstained with hematoxylin, routinely dehydrated, and sealed with neutral gel.

### CRISPR/Cas9 establishment of S100A16 knockdown NRK-52E cell line

Four sgRNAs were designed on the online design tool targeting S100A16 (sgRNA1: CTC CAG CTC TGT ATA GCA GT; sgRNA2: CTG GAG CAT CTT TCG GAA AC; sgRNA3: CTG CTA TAC AGA GAG CTG GAG A; sgRNA4: TGC TGA TCT TGT TCT TGA CC), sgRNAs were ligated to px330 vector (#42230, Addgene, USA) using BasI restriction endonuclease and DNA ligase. After sequencing verification, sgRNAs cleavage efficiency was verified using T7 endonuclease 1. The two sgRNAs (sgRNA3 and sgRNA4) with high cleavage efficiency were selected and co-transfected into 1×10^6^ NRK-52E cells with pCMV-TD-Tomato (#30530, Addgene, USA) at a ratio of 5:1 using the Lonza nuclear transfector T-027 program. After 48 h, the cells were digested, and the transfected cells were divided into 10-cm culture dishes with a density of 10 ~ 15 cells per plate at 4-fold magnification. The resistant cells were screened by DMEM medium containing G418 1.5 mg/ml in 10%FBS to remove negative cells. After 7 ~ 10 days of screening, single cell colonies were collected by trypsin and cultured in 48-well plates, and subcultured in 12-well plates. One-third of the 12-well plates were used for genomic DNA extraction and identification, and the rest were frozen for further use. Then we verified it by RT-PCR: Total RNA of cells was extracted using RNA-easy reagent according to the manufacturer’s instructions. RNA was reverse transcribed into cDNA using HiScript II Q RT SuperMix for the qPCR kit. cDNA product equivalent to 200 ng of total RNA was used as a template in a 20 μl polymerase chain reaction system. In addition, the S100A16 gene was subjected to a polymerase chain reaction using Green Taq Mix. The amplification products were separated using a 2.5% agarose gel. gel with a product size of 96 bp, using GAPDH (112 bp) as an internal reference. GAPDH: Forward: TTC ACC ACC ATG GAG AAG GC; Reverse: CTC GTG GTT CAC ACC CAT CA. S100A16: Forward: GGT TGA CTC GGA GGA GAC AGG; Reverse: TCT ACC AGG ACA ACA AGT GCC.

### Cell viability and apoptosis assay

The cell viability was detected via Cell Counting Kit-8 (CCK8). The WT and S100A16^−/−^ NRK-52E cells were respectively seeded with 3000 cells per well in 96-well plate and 6 repetitive holes in each group. After 12, 24, 48, and 72 hours of culture, 10 μl CCK-8 solution was added to each well and incubated for hours. The absorbance of the cells was detected at 450 nm. TUNEL assay (Beyotime Biotechnology, Shanghai, China) was performed to determine cell apoptosis. Briefly, cells grew in six-well plates with different treatment were fixed in a 4^o^C formaldehyde solution for 24 h. Immersed in phosphate-buffered saline (PBS) containing 0.3% (v/v) Triton X-100 and incubated for 1 h with the configured TUNEL stain at 37^o^C. After washing three times with PBS, added 4’,6-Diamidino-2-phenylindole dihydrochloride (DAPI; Abcam), and after sealing the tablet was observed using a display microscope to evaluate apoptotic cells.

### RNA extraction, purification, real-time PCR analysis

Real-time PCR: Total RNA of cells was extracted using an RNA-easy reagent according to the manufacturer’s instructions. RNA was reverse transcribed into cDNA using HiScript II Q RT SuperMix for the qPCR kit. mRNA levels were measured using ChamQ SYBR qPCR Master Mix and Applied Biosystems StepOne Plus Real-Time Fluorescence Polymerase Chain Reaction System, with the housekeeping gene GAPDH or β-actin as controls, to detect the mRNA levels of the genes of interest. All reactions were performed using the following cycling parameters: 95 °C for 10 min, followed by 35 cycles of 94 °C for 15 s and 60 °C for 50 s. All fold changes relative to the control were calculated using the 2^−ΔΔCt^ method. The primer sequences were listed in Supplementary Table 4.

### Immunofluorescence staining

Cells were seeded in 24-well plates and incubated overnight for 12 h, then fixed using 4% polymethyl at room temperature for 3 h, 1% Triton X-100 for 15 min at room temperature, 5% bovine serum albumin for 30 min at room temperature. Then incubated overnight at 4 °C with the anti-S100A16 antibody, incubated with the appropriate secondary antibody for 30 min at room temperature, DAPI staining at room temperature for 2 min, and finally, images were observed using fluorescence microscopy.

### Chromatin immunoprecipitation assays

Chromatin immunoprecipitation (ChIP) assays were performed using the ChIP assay kit (Cell Signaling Technology, Billerica, USA) according to the manufacturer’s protocol. In brief, HK-2 cells were collected and fixed with 1% formaldehyde at 37 °C for 10 min, nuclei were digested using nuclease to obtain DNA fragments of appropriate length, proteins and DNA were immunoprecipitated, chromatin was uncrosslinked from magnetic beads and subjected to DNA purification. Finally, the immunoprecipitated DNA fragments were detected by agarose electrophoresis PCR. Normal rabbit IgG was used as a negative control. The primer sequences in ChIP assays were listed in Supplementary Table 5.1 and Table 5.2.

### Dual-Luciferase reporting assay

Dual luciferase reporter assays were performed according to the manufacturer’s instructions (Promega, USA). In brief, SYVN1 and S100A16 gene sequences were cloned into the pGL3 vector, and these constructs were transfected into HK-2 cells together with the HIF-1α overexpression vector and Renilla constructs at a 2:2:1 ratio. After 48 hours, luciferase activity was measured using a dual luciferase reporter assay system. The primer sequences of luciferase plasmids are listed in Supplementary Table 6.1 and Table 6.2.

### Statistical analysis

The data are presented as mean ± SEM from at least three independent experiments. Statistical analysis software GraphPad Prism 8.0 (GraphPad Software, Inc. La Jolla, CA, USA) was used to analyze the data, and a Student t-test was used to determine the differences between groups. *P* < 0.05 was considered to be statistically significant.

### Supplementary information

supplementary Figs

original western blots

## Data Availability

All data generated during this study are included in this published article and its supplementary information files.

## References

[1] Heizmann ClausW, Fritz G, Schäfer BW (2002). S100 proteins: structure functions and pathology. Front Biosci.

[2] Donato R, Cannon BR, Sorci G, Riuzzi F, Hsu K, Weber DJ (2013). Functions of S100 Proteins. Curr Mol Med..

[3] Sturchler E, Cox JA, Durussel I, Weibel M, Heizmann CW (2006). S100A16, a Novel Calcium-binding Protein of the EF-hand Superfamily. J Biol Chem..

[4] Basnet S, Vallenari EM, Maharjan U, Sharma S, Schreurs O, Sapkota D An Update on S100A16 in Human Cancer. Biomolecules. 2023;13:1070. 10.3390/biom13071070 PMC10377057 37509106

[5] Zhang W-S, Zhang R, Ge Y, Wang D, Hu Y, Qin X (2022). S100a16 deficiency prevents hepatic stellate cells activation and liver fibrosis via inhibiting CXCR4 expression. Metabolism..

[6] Jin R, Zhao A, Han S, Zhang D, Sun H, Li M (2021). The interaction of S100A16 and GRP78 actives endoplasmic reticulum stress-mediated through the IRE1α/XBP1 pathway in renal tubulointerstitial fibrosis. Cell Death Dis..

[7] Sun Y, Fan Y, Wang Z, Li M, Su D, Liu Y (2022). S100A16 promotes acute kidney injury by activating HRD1-induced ubiquitination and degradation of GSK3β and CK1α. Cell Mol Life Sci..

[8] Jiang Y, Yu X, Zhao Y, Huang J, Li T, Chen H, et al. ADAMTS19 Suppresses Cell Migration and Invasion by Targeting S100A16 via the NF-κB Pathway in Human Gastric Cancer. Biomolecules. 2021;11:561. 10.3390/biom11040561 PMC8070242 33921267

[9] Fang D, Zhang C, Xu P, Liu Y, Mo X, Sun Q (2021). S100A16 promotes metastasis and progression of pancreatic cancer through FGF19-mediated AKT and ERK1/2 pathways. Cell Biol Toxicol..

[10] Zhang R, Kan JB, Lu S, Tong P, Yang J, Xi L, et al. S100A16-induced adipogenesis is associated with up-regulation of 11 β-hydroxysteroid dehydrogenase type 1 (11β-HSD1). Biosci Rep. 2019;39:BSR20182042. 10.1042/BSR20182042 PMC6734118 31399502

[11] de Klerk E, Xiao Y, Emfinger CH, Keller MP, Berrios DI, Loconte V, et al. Loss of ZNF148 enhances insulin secretion in human pancreatic β cells. JCI Insight. 2023;8:e157572. 10.1172/jci.insight.157572 PMC10393241 37288664

[12] Sun H, Zhao A, Li M, Dong H, Sun Y, Zhang X (2020). Interaction of calcium binding protein S100A16 with myosin-9 promotes cytoskeleton reorganization in renal tubulointerstitial fibrosis. Cell Death Dis..

[13] Li L, Shen Y, Ding Y, Liu Y, Su D, Liang X (2014). Hrd1 participates in the regulation of collagen I synthesis in renal fibrosis. Mol Cell Biochem..

[14] Huang Y, Sun Y, Cao Y, Sun H, Li M, You H (2017). HRD1 prevents apoptosis in renal tubular epithelial cells by mediating eIF2α ubiquitylation and degradation. Cell Death Dis..

[15] McGettrick AF, O’Neill LAJ (2020). The Role of HIF in Immunity and Inflammation. Cell Metab..

[16] Loboda A, Jozkowicz A, Dulak J (2010). HIF-1 and HIF-2 transcription factors–similar but not identical. Mol Cells..

[17] Muto Y, Wilson PC, Ledru N, Wu H, Dimke H, Waikar SS (2021). Single cell transcriptional and chromatin accessibility profiling redefine cellular heterogeneity in the adult human kidney. Nat Commun..

[18] Chambers BE, Gerlach GF, Clark EG, Chen KH, Levesque AE, Leshchiner I, et al. Tfap2a is a novel gatekeeper of nephron differentiation during kidney development. Development. 2019;146:dev172387. 10.1242/dev.172387 PMC6633607 31160420

[19] Wang J, Ji W, Zhu D, Wang W, Chen Y, Zhang Z (2018). Tfap2b mutation in mice results in patent ductus arteriosus and renal malformation. J Surg Res..

[20] Moser M, Rüschoff J, Buettner R (1997). Comparative analysis of AP-2 alpha and AP-2 beta gene expression during murine embryogenesis. Dev Dyn..

[21] Hu Y, Zhang R, Lu S, Zhang W, Wang D, Ge Y (2023). S100 Calcium Binding Protein A16 Promotes Cell Proliferation by triggering LATS1 ubiquitin degradation mediated by CUL4A ligase to inhibit Hippo pathway in Glioma development. Int J Biol Sci..

[22] Wang N, Wang R, Tang J, Gao J, Fang Z, Zhang M (2022). Calbindin S100A16 Promotes Renal Cell Carcinoma Progression and Angiogenesis via the VEGF/VEGFR2 Signaling Pathway. Contrast Media Mol Imaging..

[23] Ou S, Liao Y, Shi J, Tang J, Ye Y, Wu F, et al. S100A16 suppresses the proliferation, migration and invasion of colorectal cancer cells in part via the JNK/p38 MAPK pathway. Mol Med Rep. 2021;23:164. 10.3892/mmr.2020.11803 PMC7789101 33355370

[24] Zhu W, Xue Y, Liang C, Zhang R, Zhang Z, Li H (2016). S100A16 promotes cell proliferation and metastasis via AKT and ERK cell signaling pathways in human prostate cancer. Tumour Biol..

[25] Kan J, Zhao C, Lu S, Shen G, Yang J, Tong P (2019). S100A16, a novel lipogenesis promoting factor in livers of mice and hepatocytes in vitro. J Cell Physiol..

[26] Raap M, Gierendt L, Kreipe HH, Christgen M (2021). Transcription factor AP-2beta in development, differentiation and tumorigenesis. Int J Cancer..

[27] Moser M, Pscherer A, Roth C, Becker J, Mücher G, Zerres K (1997). Enhanced apoptotic cell death of renal epithelial cells in mice lacking transcription factor AP-2beta. Genes Dev..

[28] Miao Z, Balzer MS, Ma Z, Liu H, Wu J, Shrestha R (2021). Single cell regulatory landscape of the mouse kidney highlights cellular differentiation programs and disease targets. Nat Commun..

[29] Xu Q, Krause M, Samoylenko A, Vainio S. Wnt Signaling in Renal Cell Carcinoma. Cancers (Basel). 2016;8;57. 10.3390/cancers8060057 PMC4931622 27322325

[30] Wu M, Chen W, Miao M, Jin Q, Zhang S, Bai M (2021). Anti-anemia drug FG4592 retards the AKI-to-CKD transition by improving vascular regeneration and antioxidative capability. Clin Sci (Lond)..

[31] Fu Z-J, Wang Z-Y, Xu L, Chen X-H, Li X-X, Liao W-T (2020). HIF-1α-BNIP3-mediated mitophagy in tubular cells protects against renal ischemia/reperfusion injury. Redox Biol..

[32] Wei X, Hou Y, Long M, Jiang L, Du Y (2022). Molecular mechanisms underlying the role of hypoxia-inducible factor-1 α in metabolic reprogramming in renal fibrosis. Front Endocrinol (Lausanne)..

[33] Shu S, Wang Y, Zheng M, Liu Z, Cai J, Tang C, et al. Hypoxia and Hypoxia-Inducible Factors in Kidney Injury and Repair. Cells. 2019;8:207. 10.3390/cells8030207 PMC6468851 30823476

[34] Cramer T, Yamanishi Y, Clausen BE, Förster I, Pawlinski R, Mackman N (2003). HIF-1alpha is essential for myeloid cell-mediated inflammation. Cell..

[35] Luo L, Luo G, Fang Q, Sun Z (2014). Stable expression of hypoxia-inducible factor-1α in human renal proximal tubular epithelial cells promotes epithelial to mesenchymal transition. Transplant Proc..

[36] Higgins DF, Kimura K, Bernhardt WM, Shrimanker N, Akai Y, Hohenstein B (2007). Hypoxia promotes fibrogenesis in vivo via HIF-1 stimulation of epithelial-to-mesenchymal transition. J Clin Invest..

[37] Kimura K, Iwano M, Higgins DF, Yamaguchi Y, Nakatani K, Harada K (2008). Stable expression of HIF-1alpha in tubular epithelial cells promotes interstitial fibrosis. Am J Physiol Renal Physiol..

[38] Zhu Q, Wang Z, Xia M, Li P-L, Van Tassell BW, Abbate A (2011). Silencing of hypoxia-inducible factor-1α gene attenuated angiotensin II-induced renal injury in Sprague-Dawley rats. Hypertension..

[39] Wang Z, Zhu Q, Li P-L, Dhaduk R, Zhang F, Gehr TW (2014). Silencing of hypoxia-inducible factor-1α gene attenuates chronic ischemic renal injury in two-kidney, one-clip rats. Am J Physiol Renal Physiol..

[40] Liu J, Wei Q, Guo C, Dong G, Liu Y, Tang C, et al. Hypoxia, HIF, and Associated Signaling Networks in Chronic Kidney Disease. Int J Mol Sci. 2017;18:950. 10.3390/ijms18050950 PMC5454863 28468297

[41] Hara S, Kawasaki S, Yoshihara M, Winegarner A, Busch C, Tsujikawa M (2019). Transcription factor TFAP2B up-regulates human corneal endothelial cell-specific genes during corneal development and maintenance. J Biol Chem..

[42] Ka SO, Hwang HP, Jang JH, Hyuk Bang I, Bae UJ, Yu HC (2015). The protein kinase 2 inhibitor tetrabromobenzotriazole protects against renal ischemia reperfusion injury. Sci Rep..

