# Supplementary material for: HIF-1α participates in the regulation of S100A16-HRD1-GSK3β/CK1α pathway in renal hypoxia injury
Source: Cell Death Dis. 2024 May 6;15(5):316. doi: 10.1038/s41419-024-06696-5 (PMC11074340; doi:10.1038/s41419-024-06696-5)
Supplement: Supplementary file 1 — supplementary Figs [file 41419_2024_6696_MOESM1_ESM.pdf]

Supplementary Data

Supplementary Figure 1. (A) Tubular injury score in WT mice and S100A16$^{+/-}$ mice after IRI. 10 high-power fields in every group were chosen. ****P<0.0001, **P<0.01. (B) Immunohistochemical (IHC) staining of HRD1 in the WT and S100A16$^{+/-}$ mice after IRI. Scale bar: 50μm. (C) Semi-quantitative analysis of IHC staining for HIF-1α, HRD1 and S100A16 protein expression in WT mice and S100A16$^{+/-}$ IRI kidneys compared with wild type sham groups. ***P<0.001, **P<0.01, *P<0.05.

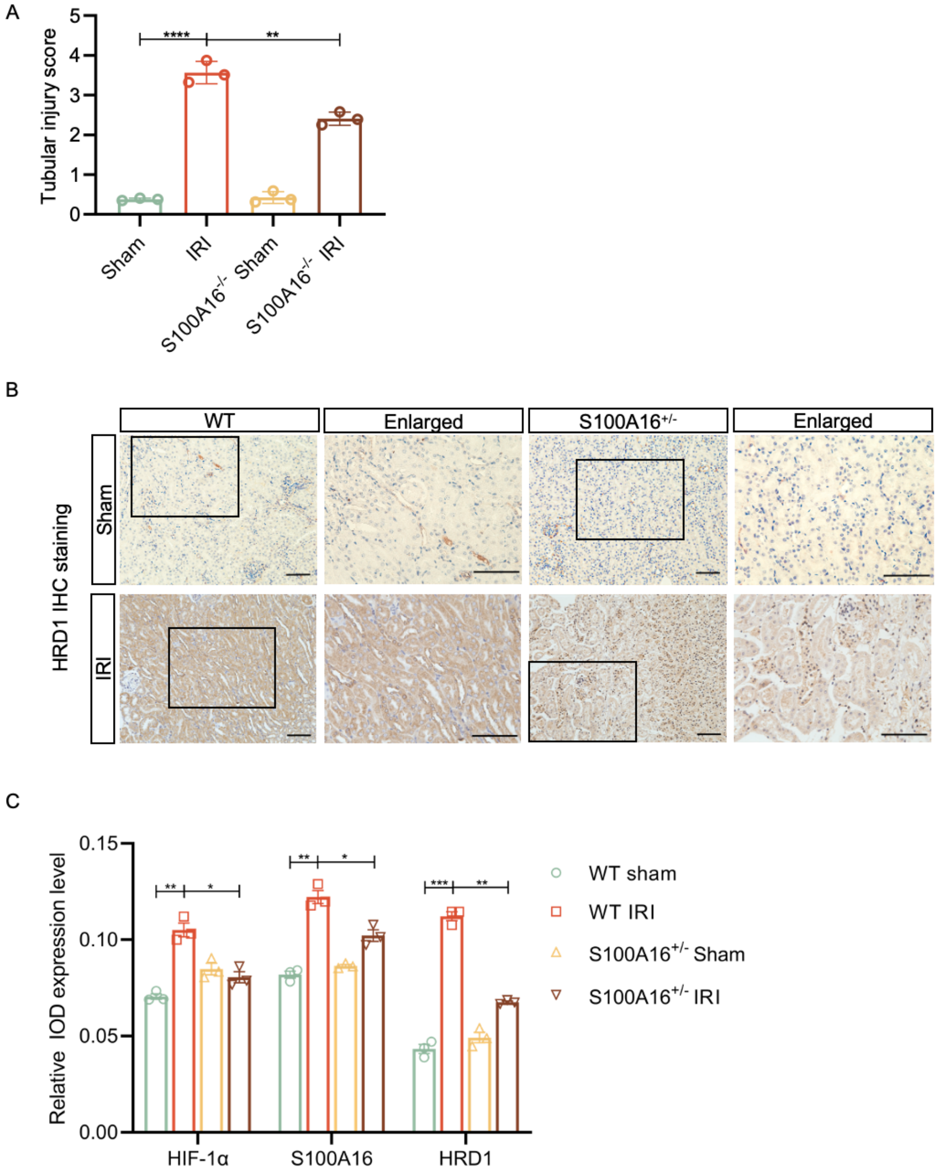

Supplementary Figure 2. Generation of S100A16 knockout NRK-52E cells. (A) T7E1 cleavage assay. M, 100 bp DNA Ladder Plus. (B) The targeting efficiency of CRISPR/Cas9 vectors detection via sequence analysis. (C) All monoclonal cell lines after screening totaled 33, and 19 positive cell lines were identified by sequencing. (D) Cellular immunofluorescence detection of S100A16 expression in WT and S100A16$^{-/-}$ cells. Scale bar: 100 μm.

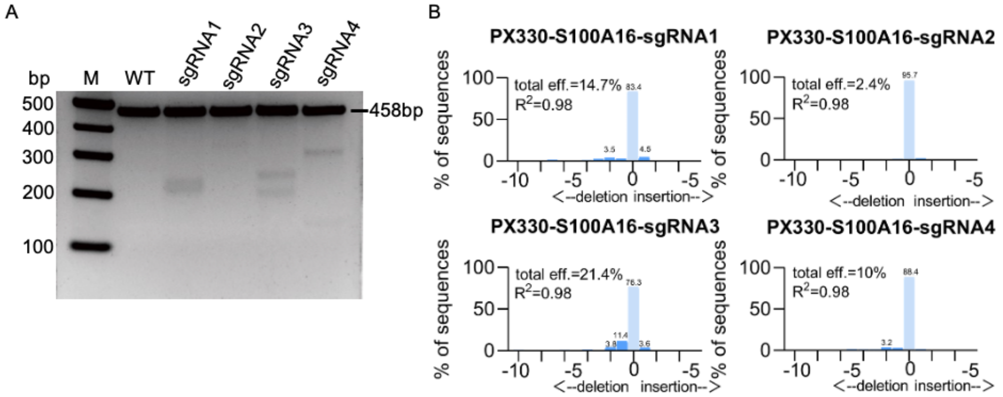

C

Monoclonal cell NO.	Sequencing results (5'-3')	Deletion
WT	ATGGCCGACTGCTATACAGAGCTGGAGAAGG======CCTGGTCAAGAACAAGATCAGCAAGTCCAG	110bp
1/3/15/17/21/25/29/32/33/36/40/41/42/43	ATGGCCGACTGCTATACAGAGCTGG---------------//-----------------CAAGAACAAGATCAGCAAGTCCAG	-61bp
19/20	ATGGCCGACTGCTATACAGAGCTG----------------//-----------------CAAGAACAAGATCAGCAAGTCCAG	-62bp
30	ATGGCCGACTGCTATACAGAGCTGGA------------------//---------------------ACAAGATCAGCAAGTCCAG	-65bp
16	A-----------------------------------//---CAAGAACAAGATCAGCAAGTCCAG	-85bp

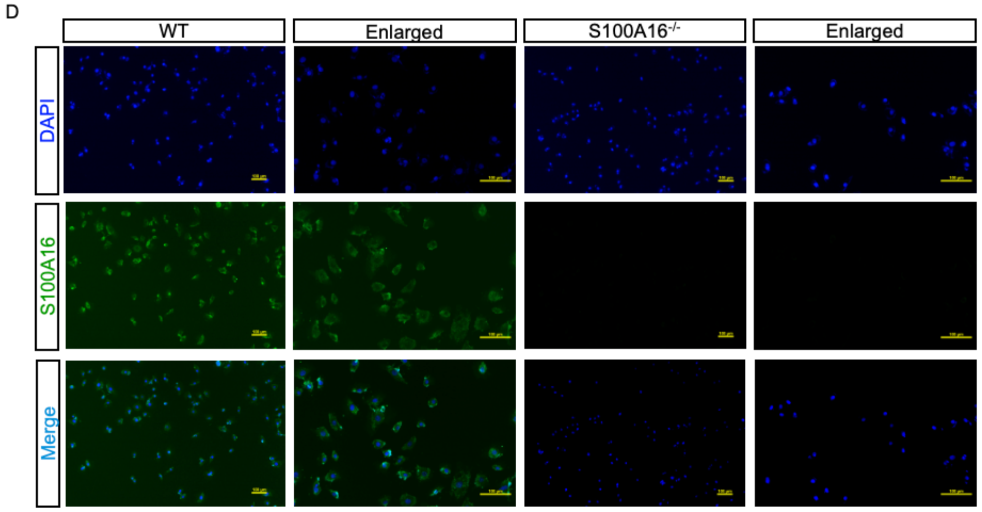

Supplementary Figure 3. Verification of S100A16 gene knockout in NRK-52E cells. (A) Microscopic morphology of WT and S100A16$^{-/-}$ NRK-52E cells. Scale bar: 100 μm. (B) The cell viability analysis in WT and S100A16$^{-/-}$ NRK-52E cells. (C) TUNEL staining and TUNEL positive cells in WT and S100A16$^{-/-}$ NRK-52E cells under the H/R conditions. ***P<0.001. (D) Western blot of Fibronectin, α-SMA and S100A16 in S100A16$^{-/-}$ cells treated with or without TGF-β1. (E) Quantitation of the western blot data in (D). **P<0.01, *P<0.05.

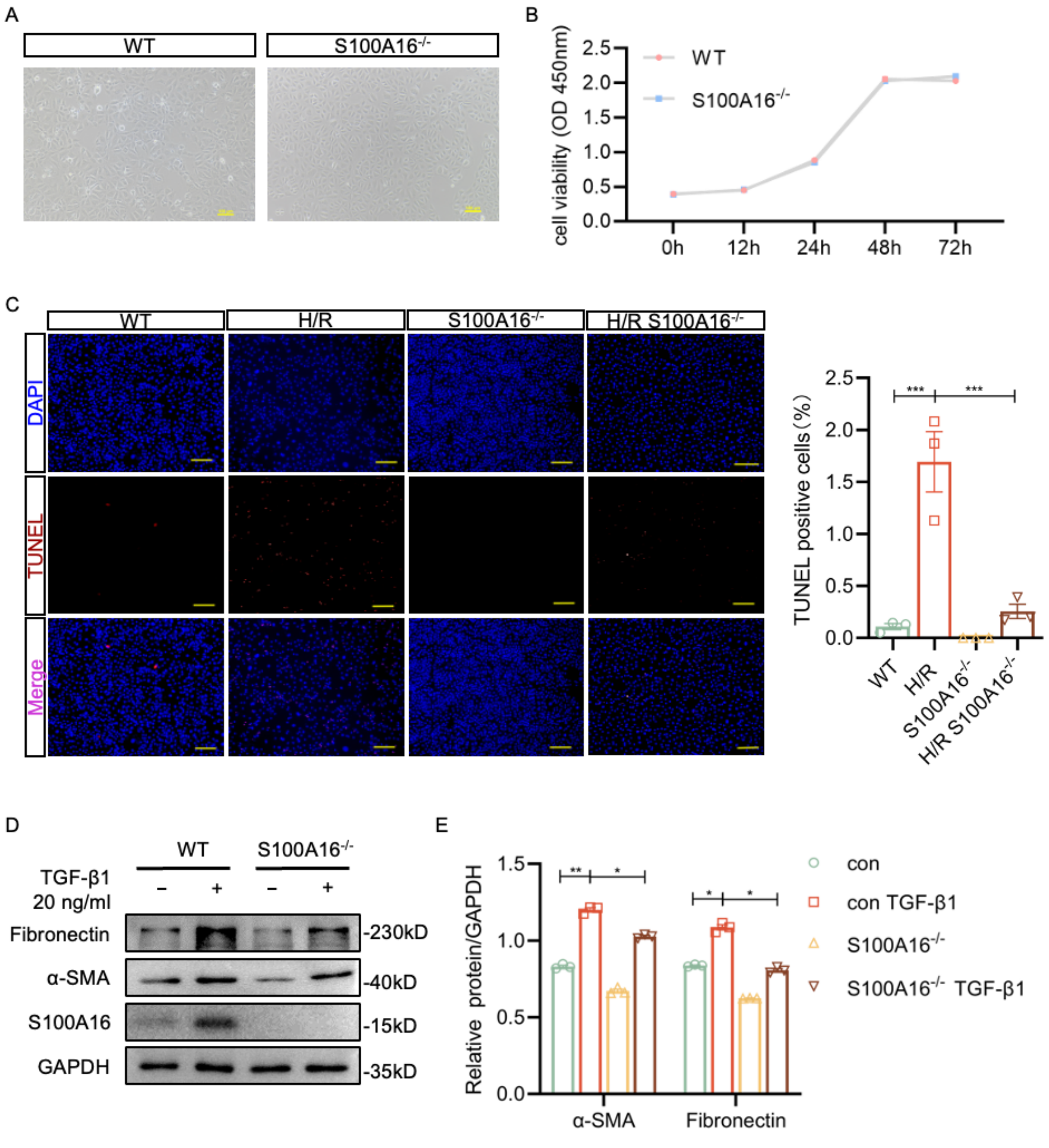

Supplementary Figure 4. (A) Western blots of S100A16, HIF-1α and HRD1 in control NRK-52E cells and S100A16^{OE} NRK-52E cells treated with or without BAY, a HIF-1α inhibitor. (B) Quantitation of the expression of S100A16, HIF-1α and HRD1 in (A). ***P<0.001, **P<0.01; *P<0.05.

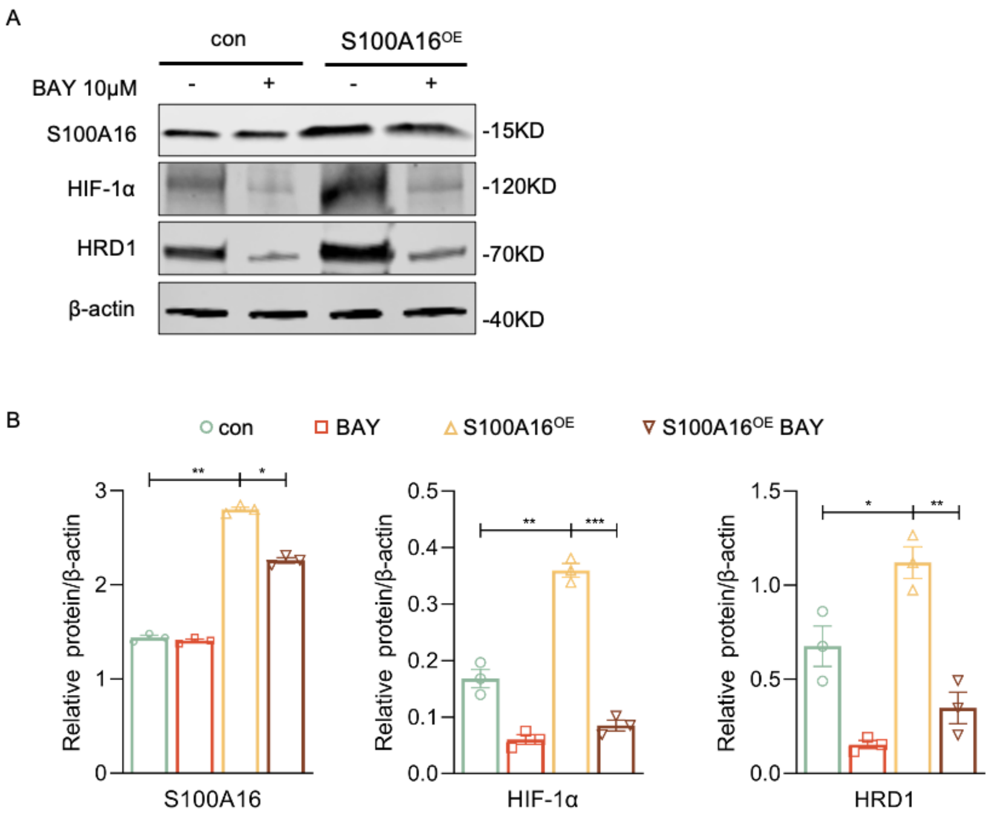

Supplementary Figure 5. (A) Western blots of TFAP2B and S100A16 in HK-2 cells treansfected with or without TFAP2B siRNA under the H/R conditions. (B) Quantitation of the expression of TFAP2B and S100A16 in (A). **P<0.01; *P<0.05. (C) Semi-quantitative analysis of IHC staining for TFAP2B protein expression in mice UUO kidneys compared with sham groups. ****P<0.0001, ***P<0.001, **P<0.01.

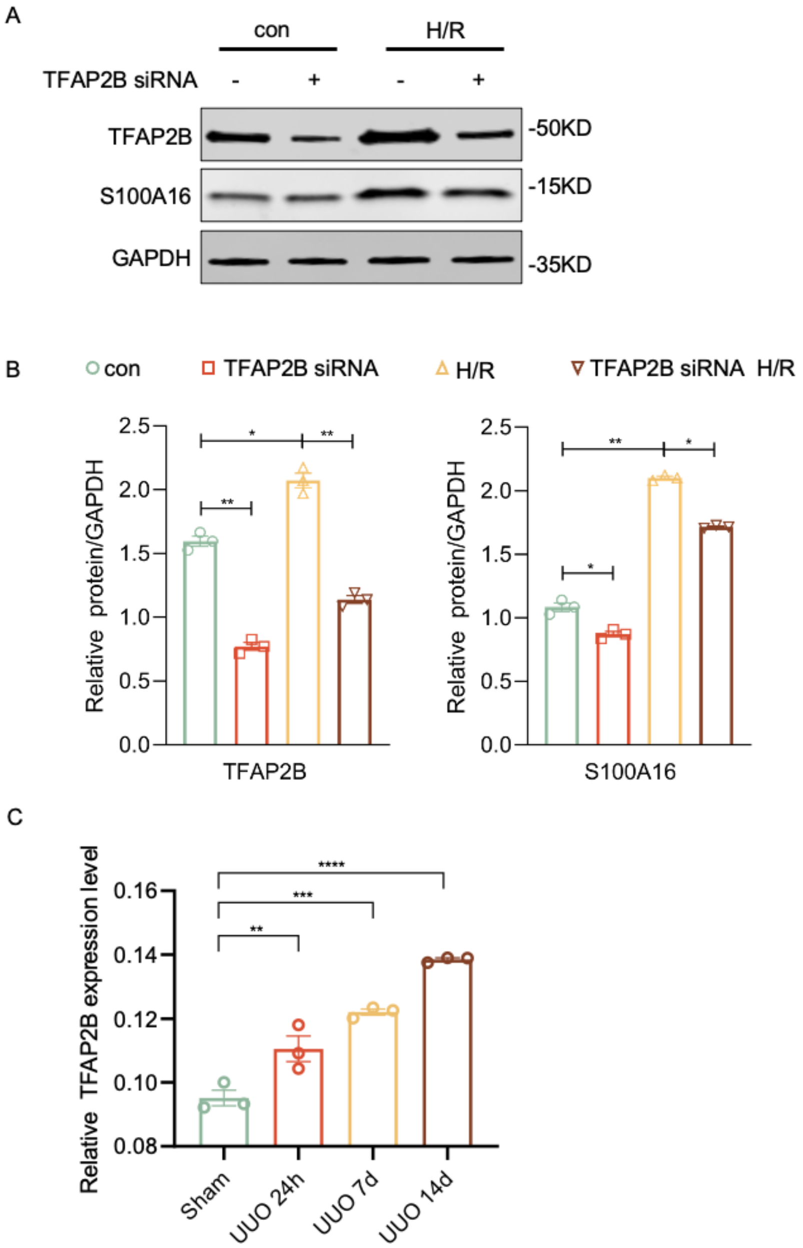

Supplementary Table 1.

1.1 JASPAR analysis of HIF-1α binding sites in the promoter of SYVN1 gene, and 5 putative site(s) were predicted with relative profile score threshold 85%.

Name	Score	Relative score	Position	Predicted sequence
HIF-1α	9.072889	0.936571	-143~-136	CTACGTGG
HIF-1α	9.024874	0.935139	-1312~-1305	ATACGTGG
HIF-1α	7.6062136	0.892817	-1144~-1137	AAACGTGG
HIF-1α	7.5398693	0.890838	-96~-89	CGGCGTGG
HIF-1α	7.357862	0.885408	-1490~-1483	AGGCGTGA

1.2 JASPAR analysis of TFAP2B binding sites in the promoter of S100A16 gene, and 2 putative site(s) were predicted with relative profile score threshold 80%.

Name	Score	Relative score	Position	Predicted sequence
TFAP2B	10.0446	0.86953	-129~-117	AGCCCCGCAGGGG
TFAP2B	9.76734	0.86540	-1893~-1881	TCCCCTCAGAGCA

Supplementary Table 2.

The expression of TFAP2B in CKD was analyzed in GEO database (GSE66494).

Sample	Title	Value
GSM1623299	Discovery_CKD_01	25.8514
GSM1623300	Discovery_CKD_02	14.0692
GSM1623301	Discovery_CKD_03	24.7297
GSM1623302	Discovery_CKD_04	36.2915
GSM1623303	Discovery_CKD_05	60.7681
GSM1623304	Discovery_CKD_06	9.63975
GSM1623305	Discovery_CKD_07	23.3221
GSM1623306	Discovery_CKD_08	11.9317
GSM1623307	Discovery_CKD_09	13.5348
GSM1623308	Discovery_CKD_10	22.466
GSM1623309	Discovery_CKD_11	8.04785
GSM1623310	Discovery_CKD_12	25.6877
GSM1623311	Discovery_CKD_13	7.73045
GSM1623312	Discovery_CKD_14	6.09056
GSM1623313	Discovery_CKD_15	10.0078
GSM1623314	Discovery_CKD_16	31.7927
GSM1623315	Discovery_CKD_17	3.418
GSM1623316	Discovery_CKD_18	23.6671
GSM1623317	Discovery_CKD_19	12.8436
GSM1623318	Discovery_CKD_20	13.8788
GSM1623319	Discovery_CKD_21	7.92448
GSM1623320	Discovery_CKD_22	10.7338
GSM1623321	Discovery_CKD_23	22.0233
GSM1623322	Discovery_CKD_24	6.45828
GSM1623323	Discovery_CKD_26	13.7949
GSM1623324	Discovery_CKD_27	13.552
GSM1623325	Discovery_CKD_28	43.9426
GSM1623326	Discovery_CKD_29	23.3126
GSM1623327	Discovery_CKD_30	38.4939
GSM1623328	Discovery_CKD_31	7.03116
GSM1623329	Discovery_CKD_32	27.1193

GSM1623330	Discovery_CKD_33	9.94252
GSM1623331	Discovery_CKD_34	9.49235
GSM1623332	Discovery_CKD_35	29.6572
GSM1623333	Discovery_CKD_36	3.59538
GSM1623334	Discovery_CKD_37	5.95228
GSM1623335	Discovery_CKD_38	18.247
GSM1623336	Discovery_CKD_39	45.4958
GSM1623337	Discovery_CKD_40	16.9071
GSM1623338	Discovery_CKD_41	25.1512
GSM1623339	Discovery_CKD_42	19.9464
GSM1623340	Discovery_CKD_43	32.1711
GSM1623341	Discovery_CKD_44	10.6536
GSM1623342	Discovery_CKD_45	15.6837
GSM1623343	Discovery_CKD_46	25.6709
GSM1623344	Discovery_CKD_47	30.1576
GSM1623345	Discovery_CKD_48	45.2094
GSM1623346	Discovery_CKD_49	13.5779
GSM1623352	Validation_CKD_01	10.5572
GSM1623353	Validation_CKD_02	34.9561
GSM1623354	Validation_CKD_03	19.7183
GSM1623355	Validation_CKD_04	32.3036
GSM1623356	Validation_CKD_05	41.191
GSM1623347	Discovery_Control_01	8.5367
GSM1623348	Discovery_Control_02	8.83453
GSM1623349	Discovery_Control_03	8.26905
GSM1623350	Discovery_Control_04	8.87078
GSM1623351	Discovery_Control_05	8.75828
GSM1623357	Validation_Control_01	10.5698
GSM1623358	Validation_Control_02	9.99479
GSM1623359	Validation_Control_03	9.81

Supplementary Table 3. Reagents and antibodies.

**Assay Kits**			Cat#	Conditions
	DMEM	Vicente	319-005-CL	
	DMEM/F12	Vicente	319-075-CL	
	10% fetal bovine serum (FBS)	Vicente	085-150	
	Anairopack	Mitsubishi gas chemical company	YZ-C-11	
	BAY	MCE	87-2243	
	TGF-β1	Peproptech		
	ExFect Transfection Reagent	Vazyme	T101-01	
	RNA-easy Isolation Reagent	Vazyme	R701-01	
	HiScript II Q RT SuperMix for qPCR kit (+gDNA wiper)	Vazyme	R123-01	
	ChamQ SYBR qPCR Master Mix (High ROX Premixed)	Vazyme	Q341-02	
	Green Taq Mix	Vazyme	P131-01	
	FastDigest BpiI（IIs）	Thermo	FD1014	
	Quick Ligation	New England Biolabs	M2200	
	T7 endonuclease 1	New England Biolabs	E3321	
	Amaxa™ basic nucleaofecter™ Kit Primary Fibroblasts	Lonza	VPI-1002	
	G418 Selective Antibiotic	MCE	HY-K1056	
	Cell Counting Kit-8 (CCK-8)	APExBIO	K1018	
	Hieff Trans® Liposomal Transfection Reagent	YEASEN	40802ES01	
	Dual-Luciferase Reporter Assay	Promega	E1910	
	SimpleChIP® Enzymatic Chromatin IP Kit (Agarose Beads)	Cell Signaling Technology	9002	
**Antibodies**				
	S100A16	Proteintech	11456-1-AP	1:1000

				Western<br>1:100 IF<br>1:100 IHC
	HRD1	Proteintech	13473-1-AP	1:1000<br>Western
	HIF-1α	Proteintech	20960-1-AP	1:2000<br>western<br>1:100 IHC
	Fibronectin	Sigma	F3648	1:1000<br>Western
	Bcl-2	Proteintech	26593-1-AP	1:1000<br>Western
	α-SMA	Proteintech	14395-1-AP	1:1000<br>Western
	Caspase 3	Cell Signaling Technology	9662S	1:1000<br>Western
	Bax	Cell Signaling Technology	2772S	1:1000<br>Western
	Cleaved caspase 3	Cell Signaling Technology	9664T	1:1000<br>Western
	GSK-3β	Cell Signaling Technology	9832S	1:1000<br>Western
	TFAP2B	Cell Signaling Technology	2509S	1:1000<br>Western
	Total β-catenin	BD Biosciences	610153	1:1000<br>Western
	GAPDH	Proteintech	60004	1:8000<br>Western

Supplementary Table 4. qPCR primer sequences.

	Gene Name	Primer sequence 5’-3’
Human	S100A16	Forward: ATGTCAGACTGCTACACGGAG
		Reverse: GTTCTTGACCAGGCTGTACTTAG
	HRD1	Forward: AACCCCTGGGACAACAAGG
		Reverse: GCGAGACATGATGGCATCTG
	TFAP2B	Forward: CTGCTCCTCACATGAATGCAC
		Reverse: CATCGTGCCGGTCCTCATAG
	HIF-1α	Forward: GAACGTCGAAAAGAAAAGTCTCG
		Reverse: CCTTATCAAGATGCGAACTCACA
	β-actin	Forward: AGGCCAACCGTGAAAAGATG
		Reverse: AGAGCATAGCCCTCGTAGATGG
Rat	HIF-1α	Forward: ACTCACAGTCGGACAACCTC
		Reverse: GCTGCAGTAACGTTCCAATTCC
	GAPDH	Forward: TTCACCACCATGGAGAAGGC
		Reverse: CTCGTGGTTCACACCCATCA

Supplementary Table 5.

5.1 The primer sequences in ChIP assays for SYVN1 gene promoter.

Name	Primer sequence 5'-3'
HRE1	Forward: AGGTTGAGTCAAGCAAGGGC
	Reverse: CTCATTGCAGCCTCGACCTC
HRE2	Forward: CAACAGGTGGCAGGGAAGAT
	Reverse: CCACTTTGTGCCTTGCGTTT
HRE3	Forward: CCTGTTGCCTTATTACCTTCCG
	Reverse: GGGGTCAGACACCTCACTTCC

5.2 The primers sequences in ChIP assays for S100A16 gene promoter.

Name	Primer sequence 5'-3'
S1	Forward: TAGCCTCTGCTCCTGCTGTCT
	Reverse: CCAGAGGGAGTTCTCAGTGC
S2	Forward: AGAGGGAGGCTCAGGGGAC
	Reverse: GAGGGGGTGTCCAGTCAAGA

Supplementary Table 6.

6.1 The primer sequences for the luciferase plasmid of SYVN1 gene promoter.

Name	Primer sequence 5'-3'
HRE1	Forward: ggatcttccagagatggtaccGTGAGCATGATGCCCGTTTCCG
	Reverse: ctgccgttcgacgatctcgagGGCTCAAGCGATCCTCCCACCT
HRE2	Forward: ggatcttccagagatggtaccGCTGAGGTGGGAGGATCGCTTGA
	Reverse: ctgccgttcgacgatctcgagCTGGTCCTGGGCTGCACTGTAT
HRE3	Forward: ggatcttccagagatggtaccGCCACTCAGCGCAGGAGCCATA
	Reverse: ctgccgttcgacgatctcgagGCCAGACTTCGCCCCACTCACC
Full length	Forward: ggatcttccagagatggtaccTGCTGTCACATCTACTAGGATAAGCTC
	Reverse: ctgccgttcgacgatctcgagACTCACCAGGAACCCAGCG

6.2 The primer sequences for the luciferase plasmid of S100A16 gene promoter.

Name	Primer sequence 5'-3'
S1	Forward: ggatcttccagagatctcgagCCTGGAATGTTAACTGAACTTGTCTG
	Reverse: ctgccgttcgacgataagcttTGACTTGGCCCTGGGGTT
Full length	Forward: ggatcttccagagatctcgagTCCCCCTTGCCAGTGCTAG
	Reverse: ctgccgttcgacgataagcttTGACTTGGCCCTGGGGTT
