# Supplementary material for: HIF-1α participates in the regulation of S100A16-HRD1-GSK3β/CK1α pathway in renal hypoxia injury
Source: Cell Death Dis. 2024 May 6;15(5):316. doi: 10.1038/s41419-024-06696-5 (PMC11074340; doi:10.1038/s41419-024-06696-5)
Supplement: Supplementary file 2 — original western blots [file 41419_2024_6696_MOESM2_ESM.pdf]

Fig. 1B

Lane1-2: WT Sham; Lane3-4: WT IRI
Lane5-6: S100A16$^{+/-}$ Sham; Lane7-8: S100A16$^{+/-}$ IRI

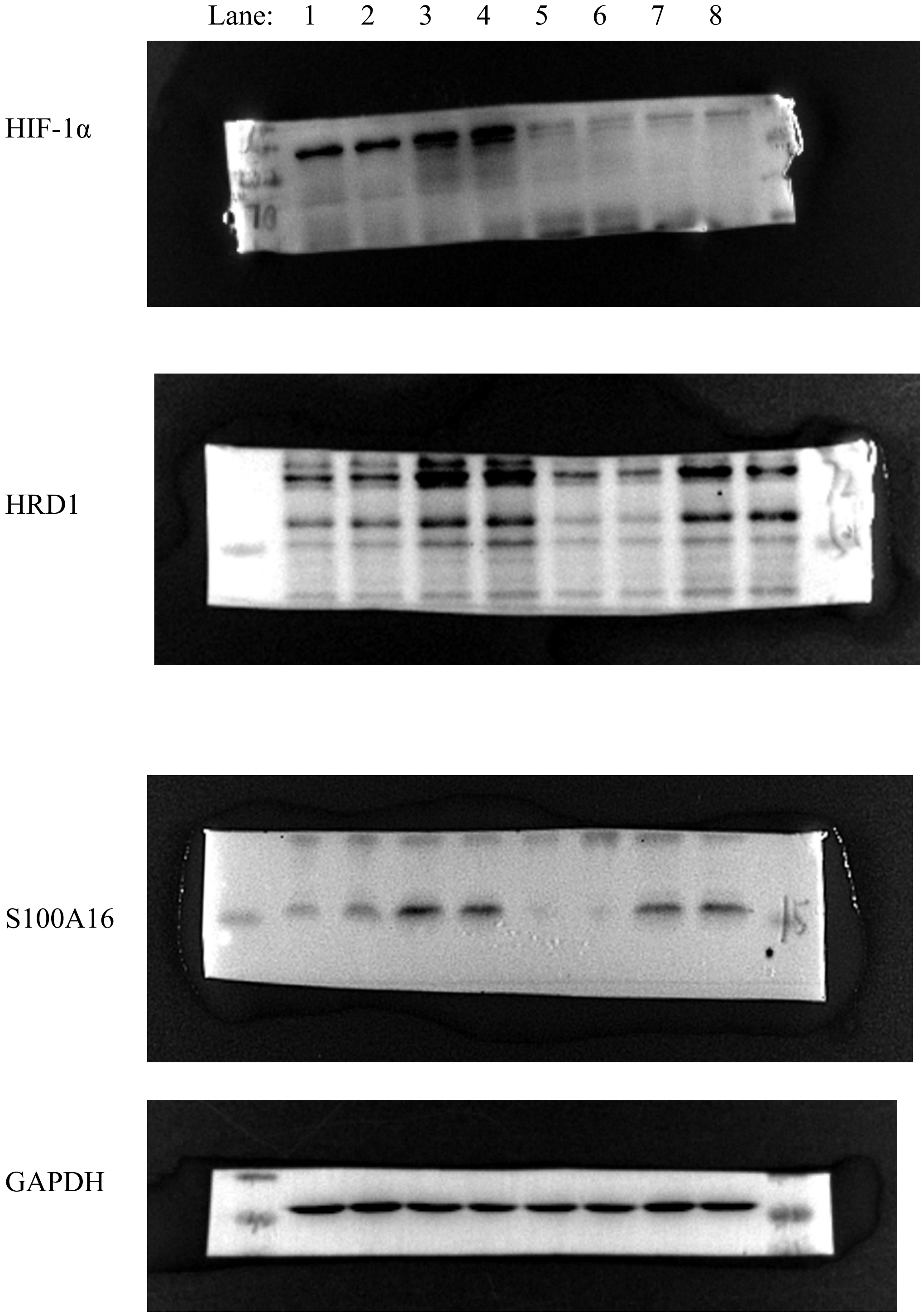

Fig. 2C

Lane 1: WT; Lane 2: S100A16$^{-/-}$ Cell NO.3
Lane 3: S100A16$^{-/-}$ Cell NO.15; Lane 4: S100A16$^{-/-}$ Cell NO.16

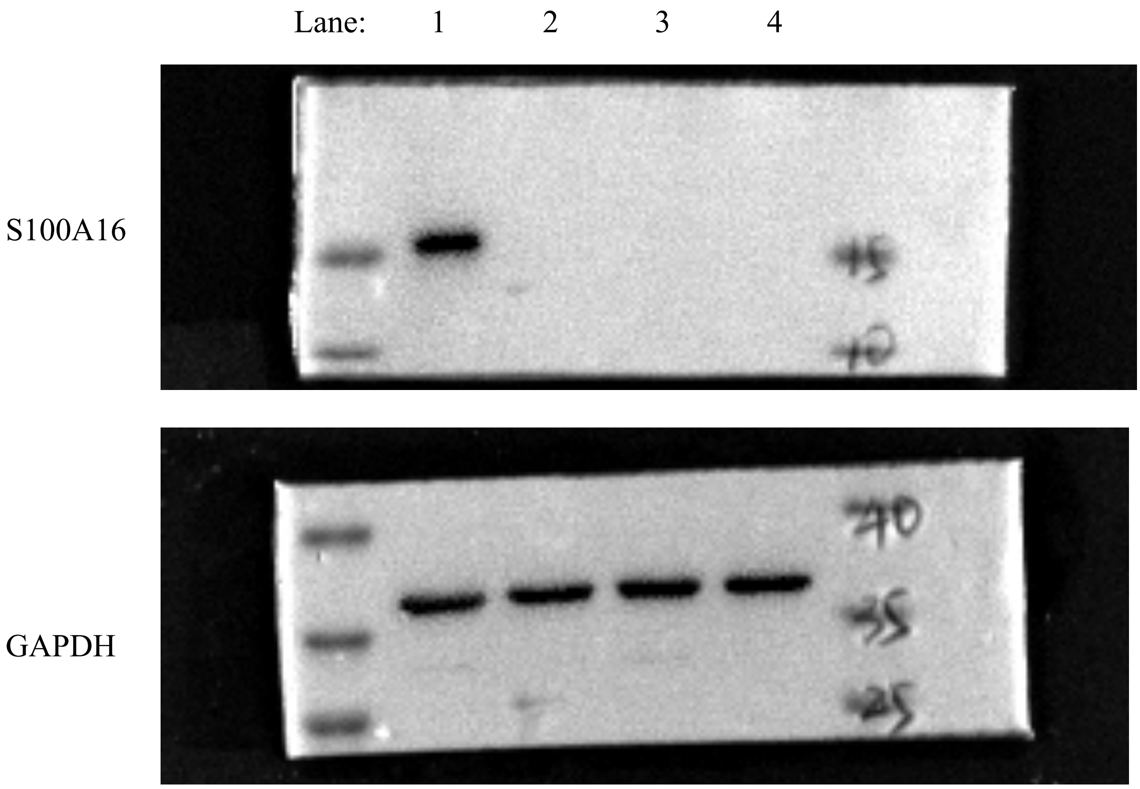

Fig. 2E

Lane1: WT; Lane2: WT H/R; Lane3: S100A16$^{-/-}$; Lane4: S100A16$^{-/-}$ H/R

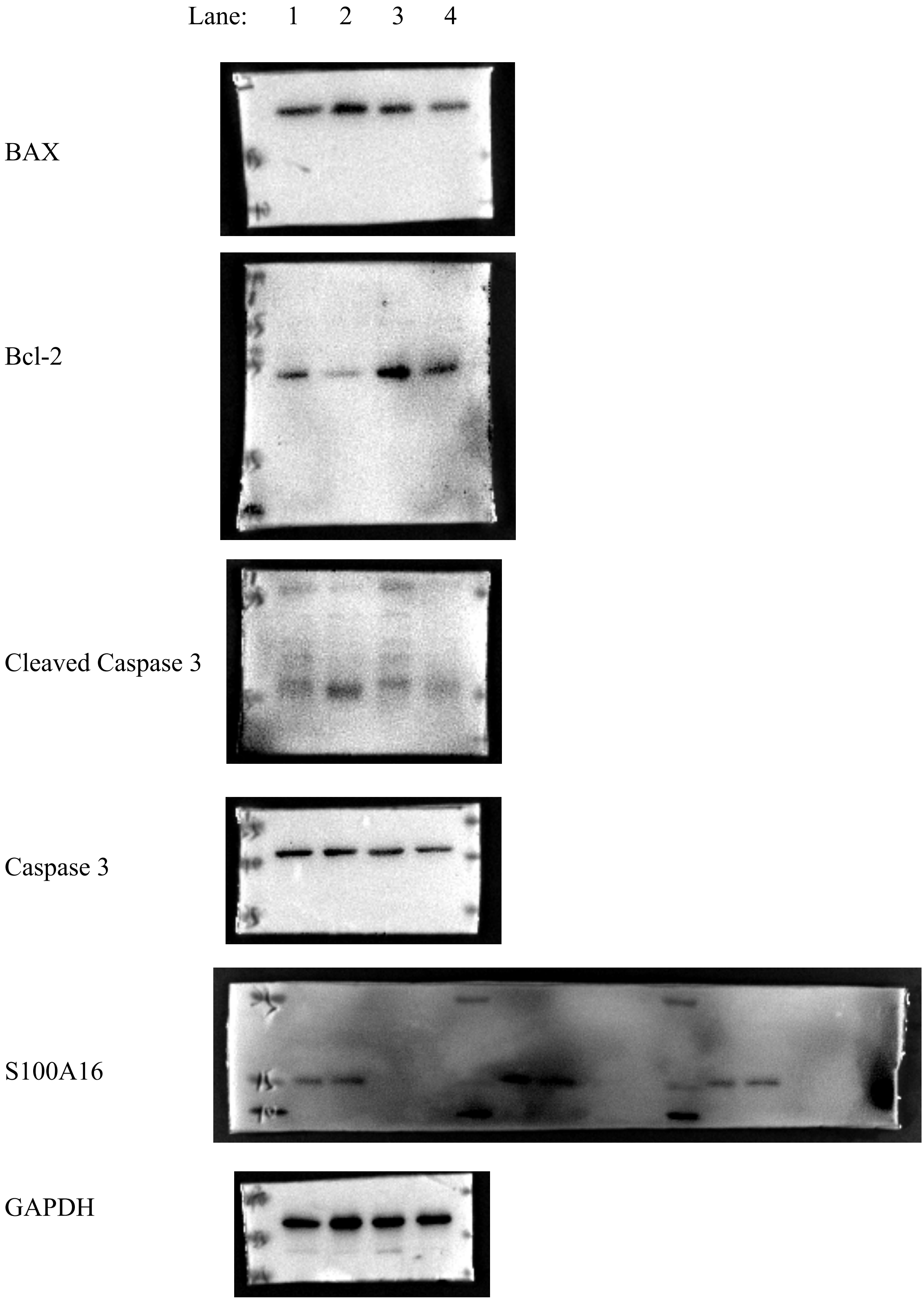

Fig. 3A

Lane1: control; Lane2: S100A16$^{-/-}$; Lane3: control H/R; Lane4: S100A16$^{-/-}$ H/R

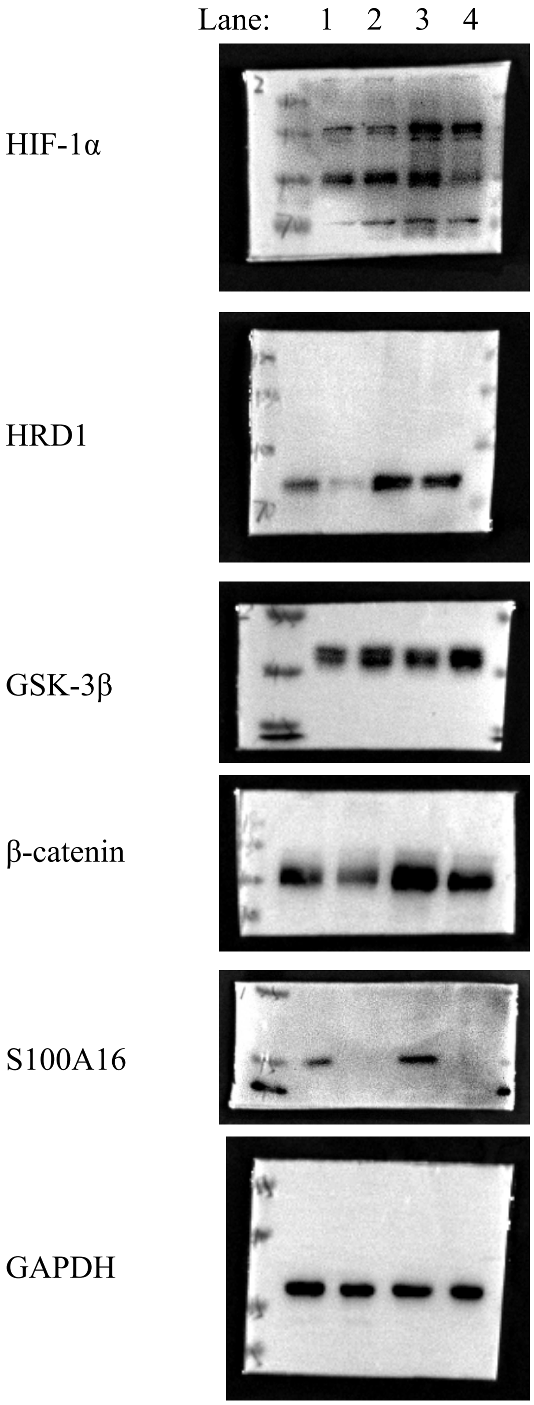

Fig. 3D

Lane1-3: NC;   Lane4-6: S100A16^{OE}

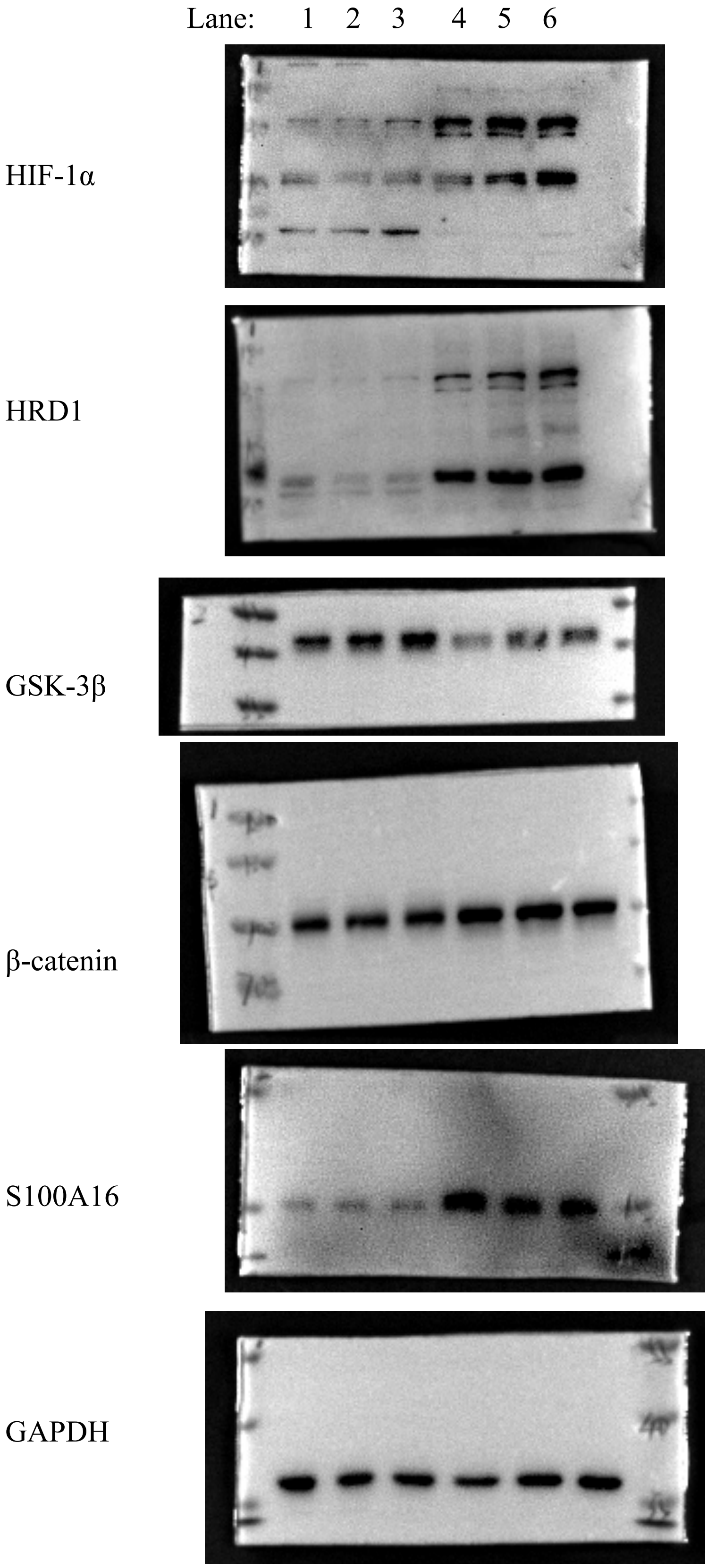

Fig. 4A

Lane1-3: NC; Lane4-6: HIF-1α^{OE}

Lane: 1 2 3 4 5 6

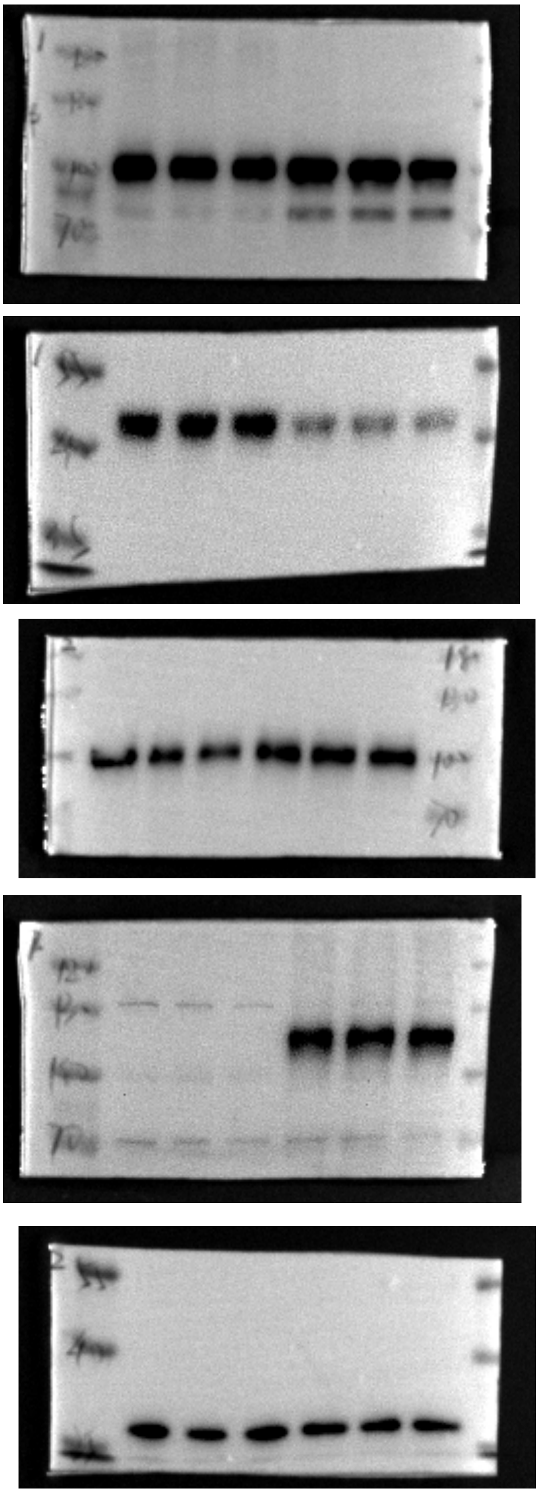

HRD1

GSK-3β

β-catenin

HIF-1α

GAPDH

Fig. 4D

Lane1: control; Lane2: H/R; Lane3: H/R BAY

Lane: 1 2 3

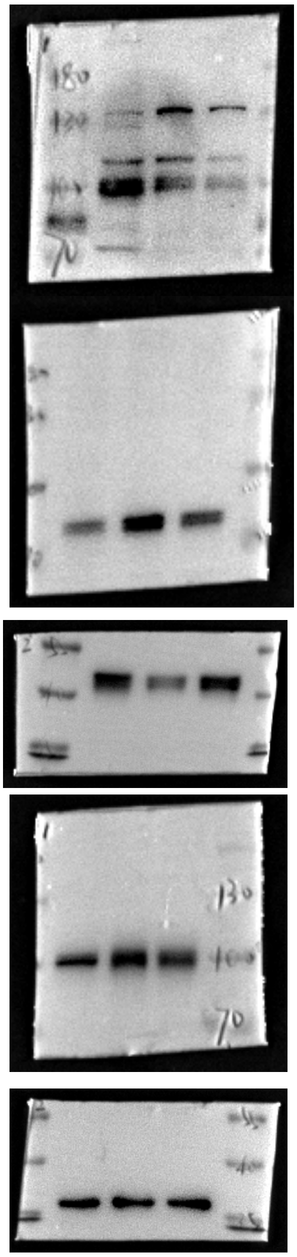

HIF-1α

HRD1

GSK-3β

β-catenin

GAPDH

Fig. 5B

Lane1-3: NC;   Lane4-6: H/R

Lane: 1 2 3 4 5 6

TFAP2B

S100A16

GAPDH

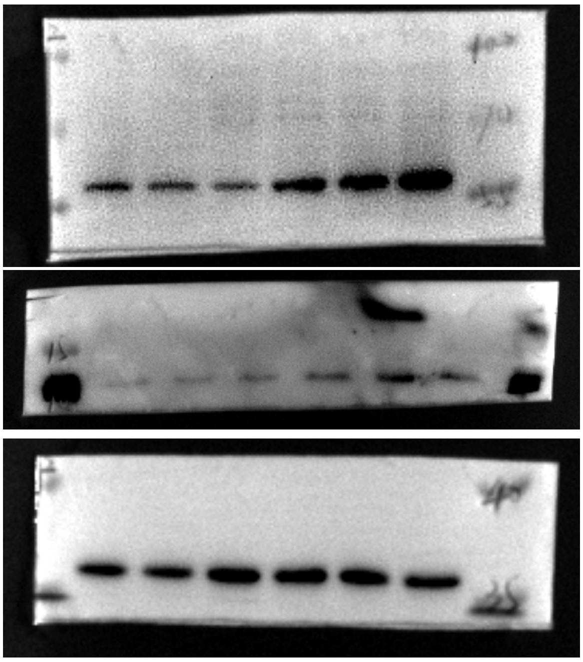

Fig. 5E

Lane1-3: NC;   Lane4-6: TGF-β1

Lane: 1 2 3 4 5 6

TFAP2B

S100A16

GAPDH

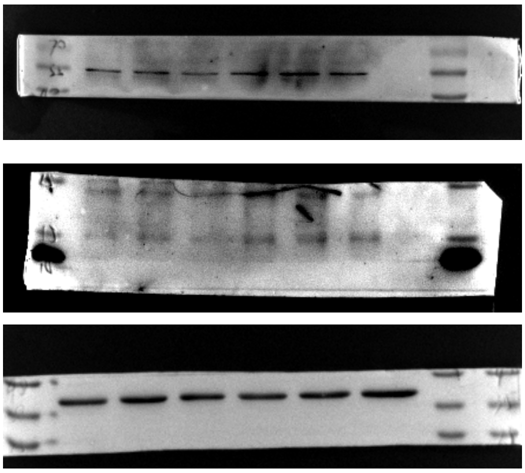

Fig. 6A

Lane1: NC; Lane2: TFAP2B^{OE}

Lane: 1 2

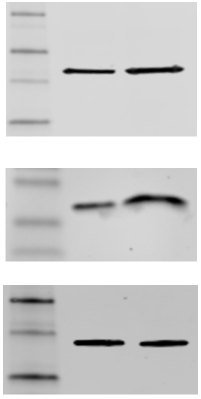

TFAP2B.

S100A16

GAPDH.

Fig. 6G

Lane1- 4: con; TFAP2B^{OE}; S100A16$^{-/-}$; S100A16$^{-/-}$ TFAP2B^{OE}

Lane: 1 2 3 4

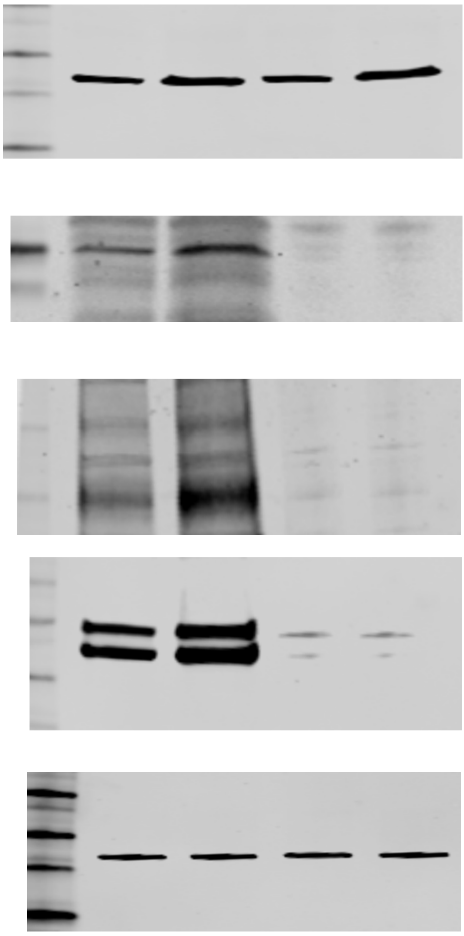

TFAP2B.

S100A16.

HIF-1α.

HRD1

β-actin.

Supplementary Fig. 3D

Lane1:WT; Lane2: WT TGF-β1; Lane3: S100A16$^{-/-}$; Lane4: S100A16$^{-/-}$ TGF-β1

Lane: 1 2 3 4

Fibronectin

α-SMA

S100A16

GAPDH

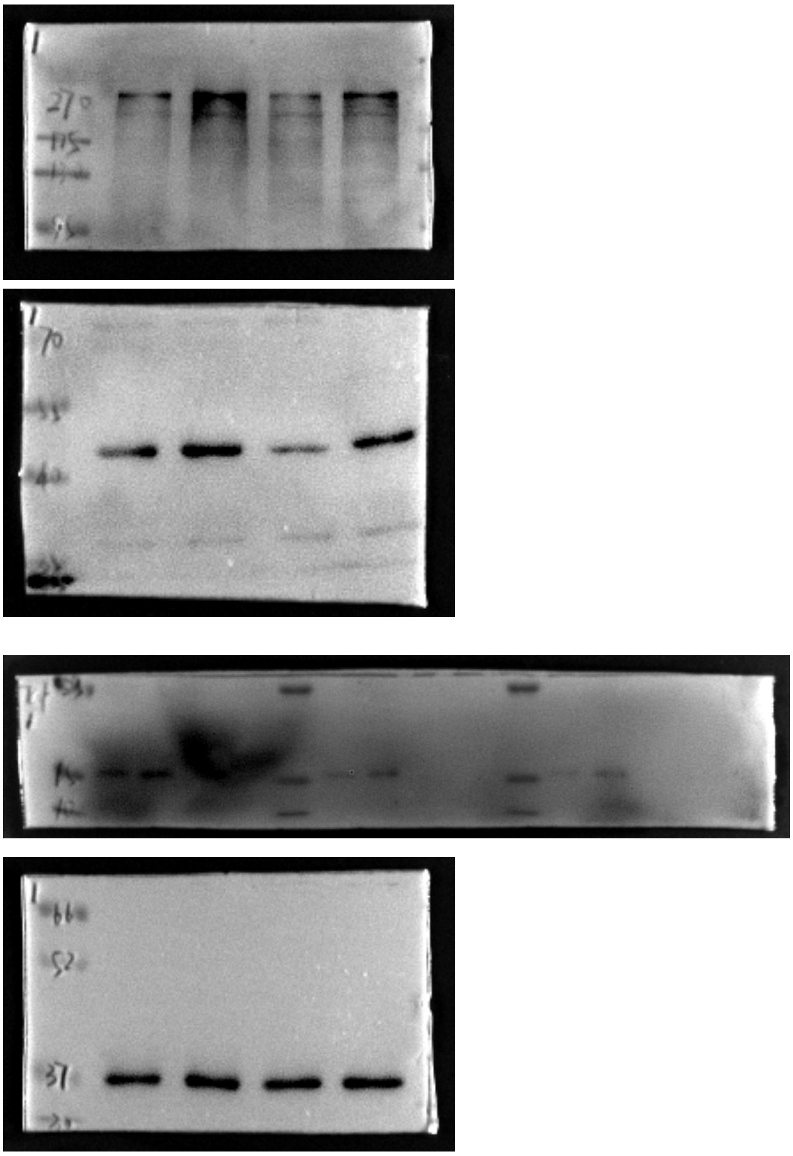

Supplementary Fig. 4A

Lane1- 4: con; BAY; S100A16^{OE}; S100A16^{OE} BAY

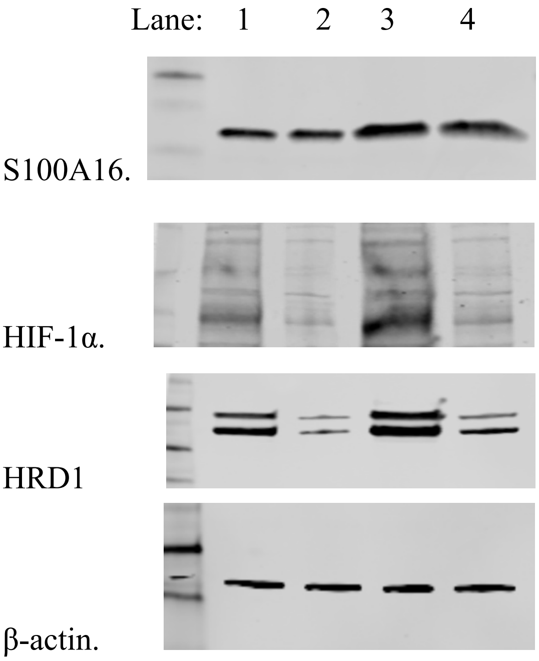

Supplementary Fig. 5A

Lane1- 4: con; TFAP2B siRNA; H/R; TFAP2B siRNA H/R

Lane: 1 2 3 4

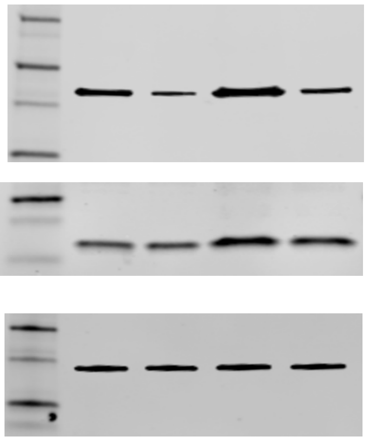

TFAP2B.

S100A16.

GAPDH.
